# Regular Use of Ivermectin as Prophylaxis for COVID-19 Led Up to a 92% Reduction in COVID-19 Mortality Rate in a Dose-Response Manner: Results of a Prospective Observational Study of a Strictly Controlled Population of 88,012 Subjects

**DOI:** 10.7759/cureus.28624

**Published:** 2022-08-31

**Authors:** Lucy Kerr, Fernando Baldi, Raysildo Lobo, Washington Luiz Assagra, Fernando Carlos Proença, Juan J Chamie, Jennifer A Hibberd, Pierre Kory, Flavio A Cadegiani

**Affiliations:** 1 General Practice, Instituto Kerr, São Paulo, BRA; 2 Animal Sciences, Universidade Estadual de São Paulo (UNESP), São Paulo, BRA; 3 Genetics, Universidade de São Paulo, Ribeirão Preto, BRA; 4 Genetics, Centro Técnico de Avaliação Genômica - C.T.A.G., Ribeirão Preto, BRA; 5 Bioinformatics, Itajaí City Hall, Itajaí, BRA; 6 Data Analysis, Universidad EAFIT, Medellín, COL; 7 Dentistry, University of Toronto, Toronto, CAN; 8 Critical Care, Front Line COVID-19 Critical Care Alliance (FLCCC), Madison, USA; 9 Clinical Endocrinology, Corpometria Institute, Brasilia, BRA; 10 Clinical Endocrinology, Applied Biology Inc, Irvine, USA

**Keywords:** ivermectin, coronavirus disease 2019, coronavirus, prevention, prophylaxis, ivermectin (ivm), rtpcr-sars-cov-2, sars-cov-2, covid-19

## Abstract

Background

We have previously demonstrated that ivermectin used as prophylaxis for coronavirus disease 2019 (COVID-19), irrespective of the regularity, in a strictly controlled citywide program in Southern Brazil (Itajaí, Brazil), was associated with reductions in COVID-19 infection, hospitalization, and mortality rates. In this study, our objective was to determine if the regular use of ivermectin impacted the level of protection from COVID-19 and related outcomes, reinforcing the efficacy of ivermectin through the demonstration of a dose-response effect.

Methods

This exploratory analysis of a prospective observational study involved a program that used ivermectin at a dose of 0.2 mg/kg/day for two consecutive days, every 15 days, for 150 days. Regularity definitions were as follows: regular users had 180 mg or more of ivermectin and irregular users had up to 60 mg, in total, throughout the program. Comparisons were made between non-users (subjects who did not use ivermectin), and regular and irregular users after multivariate adjustments. The full city database was used to calculate and compare COVID-19 infection and the risk of dying from COVID-19. The COVID-19 database was used and propensity score matching (PSM) was employed for hospitalization and mortality rates.

Results

Among 223,128 subjects from the city of Itajaí, 159,560 were 18 years old or up and were not infected by COVID-19 until July 7, 2020, from which 45,716 (28.7%) did not use and 113,844 (71.3%) used ivermectin. Among ivermectin users, 33,971 (29.8%) used irregularly (up to 60 mg) and 8,325 (7.3%) used regularly (more than 180 mg). The remaining 71,548 participants were not included in the analysis. COVID-19 infection rate was 49% lower for regular users (3.40%) than non-users (6.64%) (risk rate (RR): 0.51; 95% CI: 0.45-0.58; p < 0.0001), and 25% lower than irregular users (4.54%) (RR: 0.75; 95% CI: 0.66-0.85; p < 0.0001). The infection rate was 32% lower for irregular users than non-users (RR: 0.68; 95% CI: 0.64-0.73; p < 0.0001). Among COVID-19 participants, regularusers were older and had a higher prevalence of type 2 diabetes and hypertension than irregular and non-users. After PSM, the matched analysis contained 283 subjects in each group of non-users and regular users, between regular users and irregular users, and 1,542 subjects between non-users and irregular users. The hospitalization rate was reduced by 100% in regular users compared to both irregular users and non-users (p < 0.0001), and by 29% among irregular users compared to non-users (RR: 0.781; 95% CI: 0.49-1.05; p = 0.099). Mortality rate was 92% lower in regular users than non-users (RR: 0.08; 95% CI: 0.02-0.35; p = 0.0008) and 84% lower than irregular users (RR: 0.16; 95% CI: 0.04-0.71; p = 0.016), while irregular users had a 37% lower mortality rate reduction than non-users (RR: 0.67; 95% CI: 0.40-0.99; p = 0.049). Risk of dying from COVID-19 was 86% lower among regular users than non-users (RR: 0.14; 95% CI: 0.03-0.57; p = 0.006), and 72% lower than irregular users (RR: 0.28; 95% CI: 0.07-1.18; p = 0.083), while irregular users had a 51% reduction compared to non-users (RR: 0.49; 95% CI: 0.32-0.76; p = 0.001).

Conclusion

Non-use of ivermectin was associated with a 12.5-fold increase in mortality rate and a seven-fold increased risk of dying from COVID-19 compared to the regular use of ivermectin. This dose-response efficacy reinforces the prophylactic effects of ivermectin against COVID-19.

## Introduction

Ivermectin has been proposed as potential prophylaxis and therapy for coronavirus disease 2019 (COVID-19) due to its previously reported anti-viral [1-4], metabolic [5-10], and anti-inflammatory [11-19] actions, with strong plausibility [20,21] and positive in vitro, in vivo, and epidemiological findings [22-24] in preliminary studies.

Between July and December 2020, a citywide program in Itajaí, in the state of Santa Catarina, Southern Brazil, offered a voluntary, medically prescribed program of ivermectin as prophylaxis for COVID-19. This was based on the extensive, well-established safety profile and known absence of risks with long-term use of ivermectin, and the lack of therapeutic and preventive alternative options in 2020.

The systematically collected data within this program demonstrated that ivermectin used as prophylaxis for COVID-19 improved COVID-19 related-outcomes. The use of ivermectin led to a 44% reduction in infection rate, a 56% reduction in hospitalization rate, and a 68% reduction in mortality rate by using propensity score matching (PSM) to balance the study groups [25].

These conclusions were based on an analog evaluation of the intent-to-treat (ITT) analysis of randomized clinical trials (RCTs). All participants of the program were included for analysis, irrespective of regularity or the total amount of ivermectin taken. Among participants of the ivermectin use (regular and irregular) as prophylaxis for the COVID-19 program, it was unknown if regular ivermectin use would lead to a more substantial reduction in COVID-19 infection rate and related outcomes than irregular use.

In this study, an evaluation was done with participants that used ivermectin prophylactically for COVID-19, to determine if regular use compared to irregular use impacted the degree of reduction in COVID-19 infection, hospitalization, and mortality rates. Regular and irregular ivermectin users were also compared to non-users to evaluate evidence of a dose-response pattern of efficacy.

## Materials and methods

Study population

A thorough description of the program, study population, and protocol was described elsewhere [25]. This was a medically based, observational, and prospective study that involved the voluntary use of ivermectin as prophylaxis for COVID-19 in the city of Itajaí, Santa Catarina, Brazil. It was a citywide program conducted between July 7 and December 2, 2020. Data were collected prospectively and systematically, as was the mandatory reporting of all events.

The study design, institutional review board (IRB) approval, and data analysis were done upon completion of the program. The study of the COVID-19 cases reported in the city of Itajaí (n = 9,956, including cases that occurred before July 7, 2020, as a comparison) was approved by the National Research Ethics Council (CONEP) (approval number: 4.821.082; protocol (CAAE) number: 47124221.2.0000.5485).

Study procedures and data collection

Voluntary prophylactic use of ivermectin was offered as an option to patients during medical visits in a provisional outpatient clinic at the Convention Center and in secondary outpatient clinics at local health centers in the city of Itajaí, as part of the Universal Health System (SUS). During medical visits, patient data, including medical history, comorbidities, previous diseases, medications, and physical signs (body weight, height, body mass index, systolic and diastolic blood pressure, and heart rate), were recorded in the SUS-based system. Ivermectin was then optionally prescribed in a dose of 0.2 mg/kg/day for two consecutive days, every 15 days to participants who presented without symptoms of COVID-19 or any contradictions to ivermectin.

During the study, subjects who became infected with COVID-19 and diagnosed with a positive reverse transcription-polymerase chain reaction (RT-PCR) for SARS-CoV-2 were documented and medically followed up. Data on hospitalizations and deaths due to COVID-19 were also systematically registered.

In this analysis, all residents from the city of Itajaí were considered. This included participants in the program that used and did not use ivermectin prophylactically. Registry data were analyzed for all participants included in the sample. Subjects with a positive diagnosis of COVID-19 before July 7, 2020, when the program was initiated, and those below 18 years old were excluded from the analysis.

The 223,128 residents from Itajaí included 114,568 participants aged 18 years and above, who used ivermectin prophylactically and 45,716 who did not use ivermectin, throughout the citywide program. Among these participants, 113,844 were not infected prior to July 7, 2020. This program also included 8,352 subjects aged 18 years and above from other cities that participated in the program, although not included in the present analysis.

While ivermectin non-users remained unchanged from the first analysis [25], ivermectin users were divided according to the accumulated dose of ivermectin taken.

The analysis focused on data for participants that used up to 60 mg (10 tablets) of ivermectin and those that used more than 180 mg (more than 30 tablets). Grouping the users in this manner represented a higher certainty of regularity and irregularity, respectively. These groups were compared to non-users in a three-group comparison analysis.

The three two-group matching of ivermectin, i.e., (1) non-users and regular users, (2) non-users and irregular users, and (3) regular users and irregular users, were balanced and matched using PSM with the following variables: age, sex, history of smoking, myocardial infarction (MI), stroke, hypertension, type 2 diabetes (T2D), cardiovascular diseases (CVD), cancer (any type), asthma, chronic obstructive pulmonary disease (COPD), and other pulmonary diseases.

Because the accuracy of the reports was guaranteed for Itajaí residents only, all calculations and rates were based on the participants from the city. The database used for the calculation of COVID-19 infection rate and for risk of dying from COVID-19 was the entire city of Itajaí and then calculated among ivermectin regular users, irregular users, and non-users of participants from Itajaí. Analyses were performed before and after adjustment for multiple variables.

Hospitalization and mortality rates were analyzed for all participants reported with a positive COVID-19 diagnosis from Itajaí. Reports of all COVID-19 deaths were mandatory, while hospitalization rates were based on the data from the local public hospital only, which may justify potential discrepancies between hospitalization and mortality rates. We calculated hospitalization and hospitalization rates before matching and after PSM of groups, followed by a multivariate-adjusted analysis of the residual differences (double-adjusted model).

**Figure 1:**
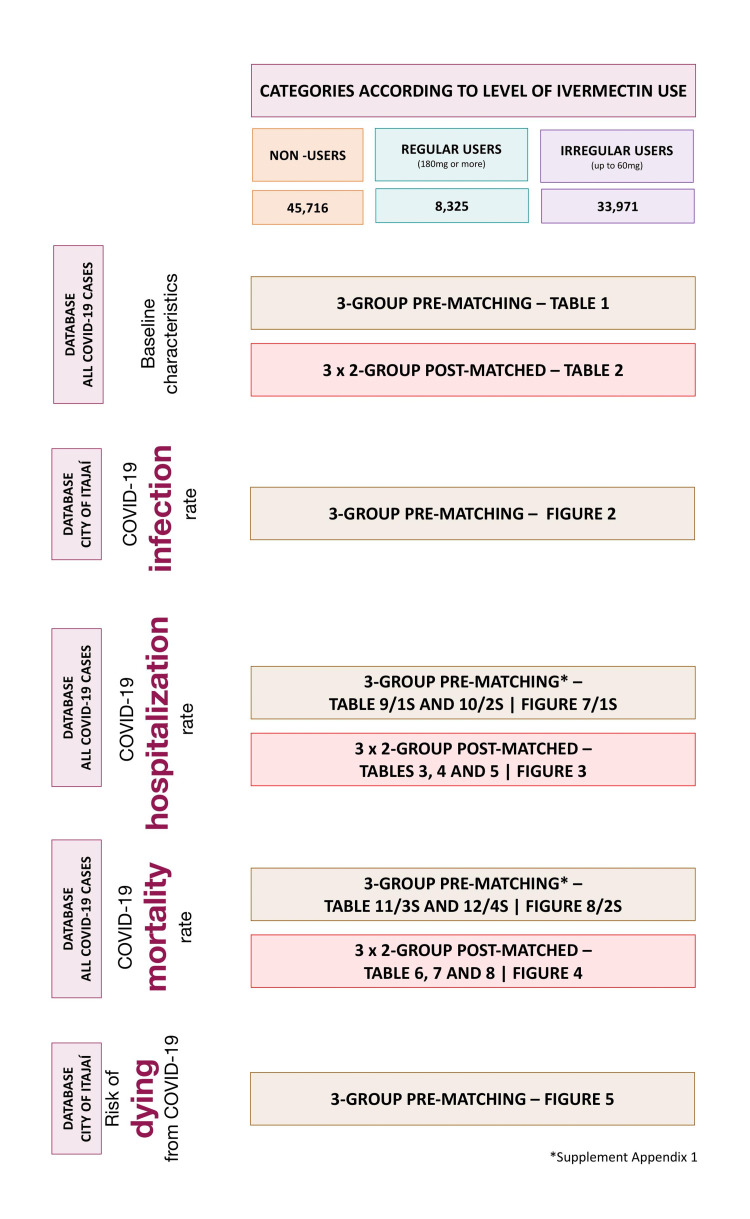
Illustrative study guide

In Supplement Appendix 1, pre-matched comparisons of hospitalization and mortality rates are provided. Figure 1 illustrates the locations of each analysis performed in this study. Datasets are publicly available at https://osf.io/uxhaf/.

Statistical analysis

The risk of hospitalizations and deaths were calculated for all three groups before matching and for each of the three two-group combinations that were propensity score matched.

Comparisons between groups for hospitalization and mortality rates were calculated using chi-square before adjusting for variables and after multivariate adjustments. The generalized linear mixed model was employed, assuming the binomial distribution for the residues and included the fixed classificatory effects for each of the variables. While there were no missing data, as per the system, illogical data, restricted to age, were corrected individually, although some may remain due to the exceptional amount of data gathered. Age between 100 and 115 years old was considered presumably illogical, rechecked, and corrected when needed. Age above 115 years old was considered obligatorily illogical and corrected accordingly. Statistical analysis software SAS/STAT (SAS Institute Inc., Cary, North Carolina, USA) was used for the present study.

This article was previously posted to the ResearchGate preprint server on July 11, 2022.

## Results

There were 159,560 participants aged 18 years and above not infected with COVID-19 prior to July 7, 2020, from the city of Itajaí, Brazil. Among them, 45,716 (28.7%) did not use ivermectin and 113,844 (71.3%) used ivermectin prophylactically. Of the 113,844 participants, 8,325 (7.3%) subjects used ivermectin regularly and 33,971 (29.8%) used ivermectin irregularly. In total, 88,012 subjects were included in the present analysis. The 71,548 (62.8%) remaining participants used intermediate doses between 60 mg and 180 mg and were not included in this analysis.

Before matching, a total of 7,228 subjects from the city of Itajaí were infected with COVID-19 between July 7 and December 2, 2020. Of these, 3,034 (42.0%) did not use ivermectin prophylactically, 283 (3.9%) used ivermectin regularly, 1,542 (21.3%) used ivermectin irregularly, and 2,369 (32.8%) used intermediate doses of ivermectin. Comparisons between ivermectin non-users, regular users, and irregular users are described in Table 1.

**Table 1:** Pre-matched baseline characteristics of ivermectin non-users, regular users, and irregular users SD = standard deviation; COPD = chronic obstructive pulmonary disease; MI = myocardial infarction.

Characteristics	Non-users (n = 3,034)	Regular users (n = 283)	Irregular users (n = 1,542)	P-value (between the three groups)
Mean (SD)	39.8 ± 14.2	47.0 ± 14.2	41.0 ± 14.5	
Age				<0.0001
<30 years	844 (27.8%)	39 (13.8%)	397 (25.7%)	
30-50 years	1,582 (52.2%)	131 (46.3%)	775 (50.3%)	
>50 years	608 (20.0%)	113 (39.9%)	370 (24.0%)	
Sex				0.19
Female	1,624 (53.5%)	141 (49.8%)	853 (55.3%)	
Male	1,410 (46.5%)	142 (50.2%)	689 (44.7%)	
Race				0.055
Afro-Brazilian	100 (3.3%)	4 (1.4%)	37 (2.4%)	
Mixed	682 (22.5%)	58 (20.5%)	373 (24.2%)	
Caucasian	2,192 (72.5%)	221 (78.1%)	1,102 (71.5%)	
Asian-Brazilian	60 (51.7%)	0 (0.0%)	30 (1.9%)	
Type 2 diabetes				0.33
Yes	63 (2.1%)	9 (3.2%)	40 (2.6%)	
No	2,971 (97.9%)	274 (96.8%)	1,502 (97.4%)	
Hypertension				0.15
Yes	166 (5.5%)	23 (8.1%)	96 (6.2%)	
No	2,868 (94.5%)	260 (91.9%)	1,446 (93.8%)	
Asthma				0.47
Yes	6 (0.2%)	0 (0.0%)	6 (0.4%)	
No	3,028 (99.8%)	283 (100.0%)	1,536 (99.6%)	
COPD				0.42
Yes	6 (0.2%)	1 (0.4%)	1 (0.1%)	
No	3,028 (99.8%)	282 (99.6%)	1,541 (99.9%)	
Other respiratory diseases				0.78
Yes	5 (0.2%)	1 (0.4%)	3 (0.2%)	
No	3,029 (99.8%)	282 (99.6%)	1,539 (99.8%)	
Cardiovascular diseases				0.11
Yes	15 (0.5%)	2 (0.7%)	16 (1.0%)	
No	3,019 (99.5%)	281 (99.3%)	1,526 (99.0%)	
Cancer				0.73
Yes	12 (0.4%)	2 (0.7%)	6 (0.4%)	
No	3,022 (99.6%)	281 (99.3%)	1,536 (99.6%)	
History of smoking				0.81
Yes	47 (1.5%)	3 (1.1%)	23 (1.5%)	
No	2,987 (98.5%)	280 (98.9%)	1,519 (98.5%)	
History of stroke				0.71
Yes	10 (0.3%)	1 (0.4%)	3 (0.2%)	
No	3,024 (99.7%)	282 (99.6%)	1,539 (99.8%)	
History of MI				0.64
Yes	4 (0.1%)	0 (0.0%)	3 (0.2%)	
No	3,030 (99,9%)	283 (100.0%)	1,539 (99.8%)	

Baseline characteristics

Table 1 describes the baseline characteristics of the groups of ivermectin non-users (n = 3,034), regular users (n = 283), and irregular users (n = 1,542) before matching groups. Age was significantly different across groups for levels of ivermectin use (p < 0.0001). Ivermectin regular users had a higher percentage of subjects above 50 years old (39.9%) than irregular users (24.0%) and non-users (20.0%). There were fewer subjects below 30 years old among regular users (13.8%) than among irregular users (25.7%) and non-users (27.8%). All other baseline characteristics were numerical but not statistically different. There were slightly more males among regular users (50.2%) than irregular users (44.7%) and non-users (46.5%) (p = 0.19). The percentage of participants with T2D was numerically higher among regular users (3.2%) than irregular users (2.6%) and non-users (2.1%) (p = 0.33). Hypertension was more prevalent in regular users (8.1%) than irregular users (6.2%) and non-users (5.5%) (p = 0.15).

Table 2 describes the baseline characteristics of ivermectin non-users paired with regular users and non-users paired with irregular users. After balancing and matching each of the three combinations of two groups (non-users and regular users, non-users and irregular users, and regular and irregular users), there were 283 subjects in each group (n = 566) between non-users and regular users and between irregular and regular users, and 1,542 (n = 3,084) between non-user and irregular users, with similar baseline characteristics.

**Table 2:** Baseline characteristics of the prophylactic study after propensity score matching between non-users and regular users, non-users and irregular users, and irregular users and regular users SD = standard deviation; COPD = chronic obstructive pulmonary disease; MI = myocardial infarction.

	Non-users paired with regular ivermectin users		Non-users paired with irregular ivermectin users		Regular users paired with irregular ivermectin users	
Variable	Non-users (n = 283)	Regular users (n = 283)	Non-users (n = 1,542)	Irregular users (n = 1,542)	Regular users (n = 283)	Irregular users (n = 283)
Age						
Mean (SD)	41.6 ± 14.8	47.0 ± 14.2	40.3 ± 14.4	41.0 ± 14.5	47.0 ± 14.2	43.8 ± 16.0
Age						
<30 years	63 (22.3%)	39 (13.8%)	410 (26.6%)	397 (25.7%)	39 (13.8%)	60 (21.2%)
30-50 years	152 (53.7%)	131 (46.3%)	808 (52.4%)	775 (50.3%)	131 (46.3%)	132 (46.4%)
>50 years	68 (24.0%)	113 (39.9%)	324 (21.0%)	370 (24.0%)	113 (39.9%)	91 (32.2%)
Sex						
Female	156 (55.1%)	141 (49.8%)	846 (54.9%)	853 (55.3%)	141 (49.8%)	155 (54.8%)
Male	127 (44.9%)	142 (50.2%)	696 (45.1%)	689 (44.7%)	142 (50.2%)	128 (45.2%)
Race						
Afro-Brazilian	9 (3.2%)	4 (1.4%)	45 (2.9%)	37 (2.4%)	4 (1.4%)	5 (1.8%)
Mixed	58 (20.5%)	58 (20.5%)	351 (22.8%)	373 (24.2%)	58 (20.5%)	68 (24.0%)
Caucasian	213 (75.3%)	221 (78.1%)	1,114 (72.2%)	1,102 (71.5%)	221 (78.1%)	209 (73.9%)
Asian-Brazilian	3 (1.1%)	0 (0.0%)	32 (2.1%)	30 (2.0%)	0	1 (0.3%)
Type 2 diabetes						
Yes	10 (3.5%)	9 (3.2%)	37 (2.4%)	40 (2.6%)	9 (3.2%)	10 (3.5%)
No	273 (96.5%)	274 (96.8%)	1,505 (97.6%)	1,502 (97.4%)	274 (96.8%)	273 (96.5%)
Hypertension						
Yes	21 (7.4%)	23 (8.1%)	86 (5.6%)	96 (6.2%)	23 (8.1%)	20 (7.1%)
No	262 (92.6%)	260 (91.9%)	1,456 (94.4%)	1,446 (93.8%)	260 (91.9%)	263 (92.9%)
Asthma						
Yes	0	0	6 (0.4%)	6 (0.4%)	0	0
No	283 (100.0%)	283 (100.0%)	1,536 (99.6%)	1,536 (99.6%)	283 (100.0%)	283 (100.0%)
COPD						
Yes	0 (0.0%)	1 (0.3%)	1 (0.1%)	1 (0.1%)	1 (0.3%)	0
No	283 (100.0%)	282 (99.7%)	1,541 (99.9%)	1,541 (99.9%)	282 (99.7%)	283 (100.0%)
Other respiratory diseases						
Yes	1 (0.3%)	1 (0.3%)	3 (0.2%)	3 (0.2%)	1 (0.3%)	0
No	282 (99.7%)	282 (99.7%)	1,539 (99.8%)	1,539 (99.8%)	282 (99.7%)	283 (100.0%)
Cardiovascular diseases						
Yes	1 (0.3%)	2 (0.7%)	9 (0.6%)	16 (1.0%)	2 (0.7%)	5 (1.8%)
No	282 (99.7%)	281 (99.3%)	1,533 (99.4%)	1,526 (99.0%)	281 (99.3%)	278 (98.2%)
Cancer						
Yes	2 (0.7%)	2 (0.7%)	6 (0.4%)	6 (0.4%)	2 (0.7%)	2 (0.7%)
No	281 (99.3%)	281 (99.3%)	1,536 (99.6%)	1,536 (99.6%)	281 (99.3%)	281 (99.3%)
History of smoking						
Yes	2 (0.7%)	3 (1.1%)	21 (1.4%)	23 (1.5%)	3 (1.1%)	1 (0.3%)
No	281 (99.3%)	286 (98.1%)	1,521 (98.6%)	1,519 (98.5%)	280 (98.9%)	282 (99.7%)
History of stroke						
Yes	1 (0.3%)	1 (0.3%)	2 (0.1%)	3 (0.2%)	1 (0.3%)	1 (0.3%)
No	282 (99.7%)	282 (99.7%)	1,540 (99.9%)	1,539 (99.8%)	282 (99.7%)	282 (99.7%)
History of MI						
Yes	0	0	1 (0.1%)	3 (0.2%)	0	0
No	283 (100.0%)	283 (100.0%)	1,541 (99.9%)	1,539 (99.8%)	283 (100.0%)	283 (100.0%)

Impact of ivermectin on infection rates in non-users, regular users, and irregular users

**Figure 2:**
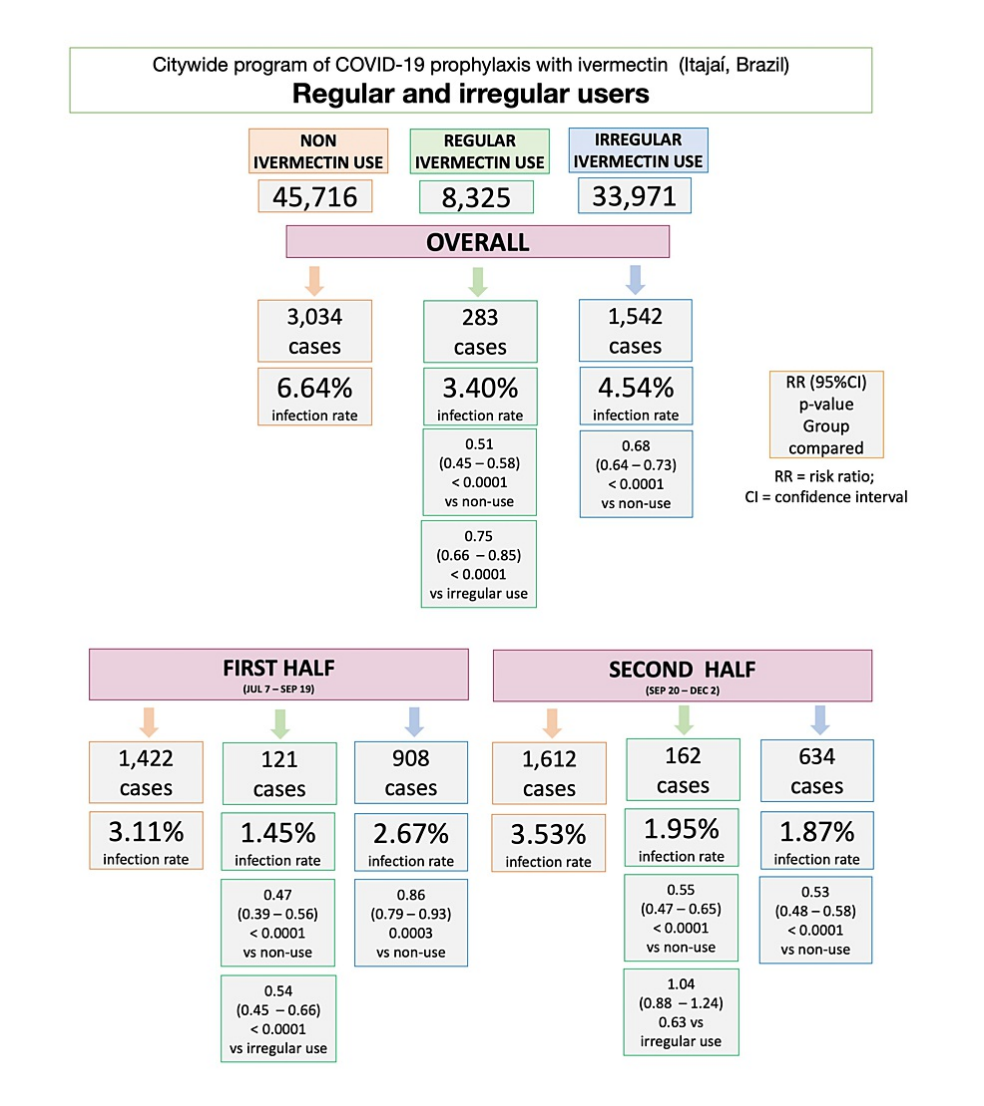
Impact of ivermectin use on infection rates during the overall, first half, and second half of the program in non-users, regular users, and irregular users

Figure 2 illustrates infection rates for ivermectin non-users, regular users, and irregular users, during the overall, first, and second half of the program. In the program, the infection rate among ivermectin non-users was 6.64% (3,034/45,716 infections). Ivermectin regular users had a reduction of 49% in infection rate compared to non-users (283/8,325 cases; 3.40% infection rate; risk ratio (RR): 0.51; 95% CI: 0.45 - 0.58; p < 0.0001). Irregular ivermectin users had a 32% lower infection rate than non-users (1,542/33,971; 4.54% infection rate; RR: 0.68; 95% CI: 0.64 - 0.73); p < 0.0001). Ivermectin regular users had a 25% lower infection rate than irregular users (RR versus sporadic users: 0.75; 95% CI: 0.66 - 0.85; p < 0.0001).

In the first half of the program, between July 7 and September 19, 2020, infection rate was 3.11% (1,422 cases) among ivermectin non-users and 1.45% (121 cases) among ivermectin regular users; a 53% reduction compared to non-users (RR: 0.47; 95% CI: 0.39 - 0.56; p < 0.0001). Infection rate was 2.67% (908 cases) among ivermectin irregular users, showing a 14% reduction compared to non-users (RR: 0.86; 95% CI: 0.79 - 0.93; p = 0.0003). Regular users had 46% lower infection rate than irregular users (RR: 0.54; 95% CI: 0.45 - 0.66; p < 0.0001).

In the second half of the program, between September 20 and December 2, 2020, infection rate was 3.53% (1,612 cases) among ivermectin non-users and 1.95% (162 cases) among ivermectin regular users; a 45% reduction compared to non-users (RR: 0.55; 95% CI: 0.47 - 0.65; p < 0.0001). Infection rate was 1.87% among ivermectin irregular users (634 cases), showing a 47% reduction in infection rate compared to non-users (RR: 0.53; 95% CI: 0.48 - 0.58; p < 0.0001). Regular users had a similar infection rate to irregular users during the second half of the program (RR: 1.04; 95% CI: 0.88 - 1.24; p = 0.63).

Hospitalization rates for ivermectin non-users, regular users, and irregular users

Supplement Appendix 1A shows hospitalization rates before matching. Tables 3-5 show hospitalization rates and unadjusted and multivariate-adjusted values for each of the three two-group comparisons after balancing and matching.

**Table 3:** Hospitalization rates in the three two-group comparisons after balancing and matching the groups of non-users and regular ivermectin users MI = myocardial infarction; COPD = chronic obstructive pulmonary disease; y/o = years old; CI = confidence interval; n/a = not applicable.

Propensity score matched non-users and regular users	Ivermectin non-users (n = 283)	Regular ivermectin users (n = 283)	Unadjusted hospital risk ratio (95% CI) and p-value (p)	Multivariate adjusted hospital risk ratio (95% CI) and p-value (p)
Overall	13/283 (4.6%)	0/283 (0.0%)	0.04 (0.002 – 0.60) (0.02)	0.00 (n/a) (<0.0001)
Age				
<30 y/o	0/63 (0.0%)	0/39 (0.0%)	1.61 (0.03 – 82.7) (0.81)	1.00 (n/a) (1.00)
30-50 y/o	3/152 (2.0%)	0/131 (0.0%)	0.16 (0.01 – 3.17) (0.23)	n/a
>50 y/o	10/68 (14.7%)	0/113 (0.0%)	0.02 (0.001 – 0.43) (0.011)	n/a (n/a) (<0.001)
Sex				
Female	7/156 (4.5%)	0/141 (0.0%)	0.07 (0.004 – 1.24) (0.07)	n/a (n/a) (<0.001)
Male	6/127 (4.7%)	0/142 (0.0%)	0.07 (0.004 – 1.18) (0.064)	n/a (n/a) (<0.001)
Race				
Afro-Brazilian	0/9 (0.0%)	0/4 (0.0%)	2/11 (0.04 – 124.5) (0.72)	1.00 (n/a) (1.00)
Mixed	3/58 (5.2%)	0/58 (0.0%)	0.14 (0.01 – 2.7) (0.19)	n/a
Caucasian	10/213 (4.7%)	0/221 (0.0%)	0.14 (0.01 – 2.68) (0.19)	n/a (n/a) (<0.001)
Asian-Brazilian	0/3 (0.0%)	0/0	n/a	n/a
Type 2 diabetes				
Yes	3/10 (30.0%)	0/9 (0.0%)	0.11 (0.005 – 2.54) (0.17)	n/a
No	10/273 (3.7%)	0/274 (0.0%)	0.05 (0.003 – 0.78) (0.033)	n/a
Hypertension				
Yes	5/21 (23.8%)	0/23 (0.0%)	0.06 (0.003 – 1.24) (0.069)	n/a
No	8/262 (3.1%)	0/260 (0.0%)	0.06 (0.003 – 1.00) (0.05)	n/a
Asthma				
Yes	0/0	0/0	n/a	n/a
No	13/283 (4.6%)	0/283 (0.0%)	0.04 (0.002 – 0.60) (0.02)	0.00 (0.00 – 0.00) (<0.001)
COPD			n/a	
Yes	0/0	0/1 (0.0%)	0.50 (0.04 – 7.10) (0.61)	n/a
No	13/283 (4.6%)	0/282 (0.0%)	0.04 (0.002 – 0.60) (0.021)	0.00 (0.00 – 0.00) (<0.001)
Other respiratory diseases				
Yes	0/1 (0.0%)	0/1 (0.0%)	1.00 (0.01 – 92.4) (1.00)	n/a
No	13/282 (4.6%)	0/282 (0.0%)	0.04 (0.002 – 0.60) (0.021)	0.00 (0.00 – 0.00) (<0.001)
Cardiovascular diseases				
Yes	0/1 (0.0%)	0/2 (0.0%)	0.33 (0.02 – 5.33) (0.44)	1.00 (n/a) (1.00)
No	13/282 (4.6%)	0/281 (0.0%)	0.04 (0.002 – 0.60) (0.021)	0.00 (0.00 – 0.00) (<0.001)
Cancer				
Yes	1/2 (50.0%)	0/2 (0.0%)	0.20 (0.01 – 8.83) (0.40)	n/a
No	12/281 (4.3%)	0/281 (0.0%)	0.04 (0.002 – 0.65) (0.024)	0.00 (0.00 – 0.00) (<0.001)
History of smoking				
Yes	0/2 (0.0%)	0/3 (0.0%)	0.71 (0.01 – 49.7) (0.88)	1.00 (n/a) (1.00)
No	13/281 (4.6%)	0/280 (0.0%)	0.04 (0.002 – 0.60) (0.021)	0.00 (0.00 – 0.00) (<0.001)
History of stroke				
Yes	0/1 (0.0%)	0/1 (0.0%)	1.00 (0.01 – 92.4) (1.00)	n/a
No	13/282 (4.6%)	0/282 (0.0%)	0.04 (0.002 – 0.60) (0.021)	0.00 (0.00 – 0.00) (<0.001)
History of MI				
Yes	0/0	0/0	n/a	n/a
No	13/283 (4.6%)	0/283 (0.0%)	0.04 (0.002 – 0.60) (0.02)	0.00 (0.00 – 0.00) (<0.001)

**Table 4:** Hospitalization rates in the three two-group comparisons after balancing and matching the groups of non-users and irregular ivermectin users MI = myocardial infarction; COPD = chronic obstructive pulmonary disease; y/o = years old; CI = confidence interval; n/a = not applicable.

Propensity score matched non-users and irregular users	Ivermectin non-users (n = 1,542)	Irregular ivermectin users (n = 1,542)	Unadjusted hospital risk ratio (95% CI) and p-value (p)	Multivariate adjusted hospital risk ratio (95% CI) and p-value (p)
Overall	47/1,542 (3.0%)	38/1,542 (2.5%)	0.80 (0.52 – 1.24) (0.32)	0.71 (0.49 – 1.05) (0.099)
Age				
<30 y/o	0/410 (0.0%)	1/397 (0.3%)	3.11 (0.13 – 76.5) (0.49)	n/a (0.98)
30-50 y/o	4/808 (0.5%)	7/775 (0.9%)	1.83 (0.53 – 6.28) (0.34)	1.82 (0.54 – 6.21) (0.34)
>50 y/o	43/324 (13.3%)	30/370 (8.1%)	0.58 (0.35 – 0.94) (0.028)	0.61 (0.39 – 0.95) (0.029)
Sex				
Female	24/846 (2.8%)	17/853 (2.0%)	0.70 (0.37 – 1.31) (0.26)	0.69 (0.38 – 1.26) (0.23)
Male	23/696 (3.3%)	21/689 (3.0%)	0.92 (0.50 – 1.68) (0.79)	0.71 (0.40 – 1.24) (0.22)
Race				
Afro-Brazilian	2/45 (4.4%)	0/37 (0.0%)	0.23 (0.01 – 4.99) (0.35)	n/a (0.98)
Mixed	9/351 (2.6%)	11/373 (3.0%)	1.15 (0.47 – 2.82) (0.75)	1.02 (0.44 – 2.34) (0.97)
Caucasian	36/1,114 (3.2%)	26/1,102 (2.4%)	0.73 (0.44 – 1.21) (0.22)	0.65 (0.40 – 1.05) (0.078)
Asian-Brazilian	0/32 (0.0%)	1/30 (3.3%)	3.31 (0.13 – 84.3) (0.47)	n/a (0.98)
Type 2 diabetes				
Yes	6/37 (16.2%)	3/40 (7.5%)	0.42 (0.097 – 1.81) (0.24)	0.51 (0.14 – 1.85) (0.31)
No	41/1,505 (2.7%)	35/1,502 (2.3%)	0.85 (0.54 – 1.35) (0.49)	0.75 (0.49 – 1.15) (0.19)
Hypertension				
Yes	13/86 (15.1%)	9/96 (9.4%)	0.58 (0.24 – 1.44) (0.24)	0.59 (0.27 – 1.31) (0.20)
No	34/1,456 (2.3%)	29/1,446 (2.0%)	0.86 (0.52 – 1.41) (0.54)	0.75 (0.47 – 1.23) (0.26)
Asthma				
Yes	0/6 (0.0%)	1/6 (16.7%)	3.55 (0.12 – 105.8) (0.47)	n/a (0.58)
No	47/1,536 (3.1%)	37/1,536 (2.4%)	0.78 (0.51 – 1.21) (0.27)	0.70 (0.46 – 1.05) (0.087)
COPD				
Yes	0/1 (0.0%)	0/1 (0.0%)	1.00 (0.01 – 92.4) (1.00)	1.00 (n/a) (1.00)
No	47/1,541 (3.0%)	38/1,541 (2.5%)	0.80 (0.52 – 1.24) (0.32)	0.71 (0.48 – 1.07) (0.11)
Other respiratory diseases				
Yes	1/3 (33.3%)	0/3 (0.0%)	0.24 (0.01 – 8.62) (0.43)	0.74 (0.00 – 1,830.4) (0.59)
No	46/1,539 (3.0%)	38/1,539 (2.5%)	0.82 (0.53 – 1.27) (0.38)	0.74 (0.49 – 1.12) (0.15)
Cardiovascular diseases				
Yes	1/9 (11.1%)	1/16 (6.3%)	0.53 (0.03 – 9.71) (0.67)	n/a (0.99)
No	46/1,533 (3.0%)	37/1,526 (2.4%)	0.80 (0.52 – 1.25) (0.33)	0.70 (0.46 – 1.06) (0.09)
Cancer				
Yes	1/6 (16.7%)	0/6 (0.0%)	0.28 (0.01 – 8.42) (0.47)	n/a (0.98)
No	46/1,536 (3.0%)	38/1,536 (2.5%)	0.82 (0.53 – 1.27) (0.38)	0.74 (0.49 – 1.11) (0.14)
History of smoking				
Yes	0/21 (0.0%)	0/23 (0.0%)	0.91 (0.02 – 48.2) (0.96)	0.97 (n/a) (1.00)
No	47/1,521 (3.1%)	38/1,519 (2.5%)	0.80 (0.52 – 1.24) (0.33)	0.71 (0.48 – 1.07) (0.10)
History of stroke				
Yes	0/2 (0.0%)	0/3 (0.0%)	0.71 (0.01 – 49.7) (0.88)	n/a (1.00)
No	47/1,540 (3.1%)	38/1,539 (2.5%)	0.80 (0.52 – 1.24) (0.32)	0.72 (0.48 – 1.08) (0.11)
History of MI				
Yes	0/1 (0.0%)	1/3 (33.3%)	1.80 (0.04 – 79.4) (0.76)	n/a (0.99)
No	47/1,541 (3.0%)	37/1,539 (2.4%)	0.78 (0.51 – 1.21) (0.27)	0.70 (0.46 – 1.07) (0.09)

**Table 5:** Hospitalization rates in the three two-group comparisons after balancing and matching the groups of regular and irregular ivermectin users MI = myocardial infarction; COPD = chronic obstructive pulmonary disease; y/o = years old; CI = confidence interval; n/a = not applicable.

Propensity score matched regular users and irregular users	Regular ivermectin users (n = 283)	Irregular ivermectin users (n = 283)	Unadjusted hospital risk ratio (95% CI) and p-value (p)	Multivariate adjusted hospital risk ratio (95% CI) and p-value (p)
Overall	0/283 (0.0%)	10/283 (3.5%)	0.05 (0.003 – 0.79) (0.034)	0.00 (n/a) (<0.0001)
Age				
<30 y/o	0/39 (0.0%)	0/60 (0.0%)	1.53 (0.03 – 78.8) (0.83)	1.00 (n/a) (1.00)
30-50 y/o	0/131 (0.0%)	3/132 (2.3%)	0.14 (0.01 – 2.75) (0.20)	n/a
>50 y/o	0/113 (0.0%)	7/91 (7.7%)	0.05 (0.003 – 0.88) (0.041)	n/a (n/a) (<0.0001)
Sex				
Female	0/141 (0.0%)	2/155 (1.3%)	0.22 (0.01 – 4.56) (0.33)	n/a (n/a) (<0.0001)
Male	0/142 (0.0%)	8/128 (6.3%)	0.05 (0.003 – 0.87) (0.04)	n/a (n/a) (<0.0001)
Race				
Afro-Brazilian	0/4 (0.0%)	0/5 (0.0%)	1.22 (0.02 – 74.3) (0.92)	1.00 (n/a) (1.00)
Mixed	0/58 (0.0%)	3/68 (4.4%)	0.16 (0.01 – 3.16) (0.23)	n/a
Caucasian	2/221 (0.0%)	7/209 (3.3%)	0.26 (0.05 – 1.28) (0.099)	n/a (n/a) (<0.0001)
Asian-Brazilian	0/0	0/1 (0.0%)	3.00 (0.02 – 473.1) (0.67)	n/a
Type 2 diabetes				
Yes	0/9 (0.0%)	1/10 (10.0%)	0.33 (0.01 – 9.26) (0.52)	n/a
No	0/274 (0.0%)	9/273 (3.3%)	0.05 (0.003 – 0.88) (0.04)	0.00 (n/a) (<0.0001)
Hypertension				
Yes	0/23 (0.0%)	1/20 (5.0%)	0.28 (0.01 – 7.18) (0.44)	n/a
No	0/260 (0.0%)	9/263 (3.4%)	0.05 (0.003 – 0.89) (0.041)	n/a (n/a) (<0.0001)
Asthma				
Yes	0/0	0/0	n/a	n/a
No	0/283 (0.0%)	10/283 (3.5%)	0.05 (0.003 – 0.79) (0.034)	0.00 (n/a) (<0.0001)
COPD				
Yes	0/1 (0.0%)	0/0	0.33 (0.002 – 52.6) (0.67)	n/a
No	0/282 (0.0%)	10/283 (3.5%)	0.05 (0.003 – 0.79) (0.034)	0.00 (n/a) (<0.0001)
Other respiratory diseases				
Yes	0/1 (0.0%)	0/0	0.33 (0.002 – 52.6) (0.67)	n/a
No	0/282 (0.0%)	10/283 (3.5%)	0.05 (0.003 – 0.79) (0.034)	0.00 (n/a) (<0.0001)
Cardiovascular diseases				
Yes	0/2 (0.0%)	0/5 (0.0%)	2.20 (0.03 – 146.0) (0.71)	1.00 (n/a) (1.00)
No	0/281 (0.0%)	10/278 (3.6%)	0.05 (0.003 – 0.78) (0.033)	0.00 (n/a) (<0.0001)
Cancer				
Yes	0/2 (0.0%)	0/2 (0.0%)	1.00 (0.01 – 73.3) (1.00)	1.00 (n/a) (1.00)
No	0/281 (0.0%)	10/281 (3.6%)	0.05 (0.003 – 0.79) (0.034)	0.00 (n/a) (<0.0001)
History of smoking				
Yes	0/3 (0.0%)	0/1 (0.0%)	0.43 (0.01 – 33.6) (0.70)	1.00 (n/a) (1.00)
No	0/280 (0.0%)	10/282 (3.5%)	0.05 (0.003 – 0.79) (0.034)	0.00 (n/a) (<0.0001)
History of stroke				
Yes	0/1 (0.0%)	0/1 (0.0%)	1.00 (0.01 – 92.4) (1.00)	n/a
No	0/282 (0.0%)	10/282 (3.5%)	0.05 (0.003 – 0.79) (0.034)	0.00 (n/a) (<0.0001)
History of MI				
Yes	0/0	0/0	n/a	n/a
No	0/283 (0.0%)	10/283 (3.5%)	0.05 (0.003 – 0.79) (0.034)	0.00 (n/a) (<0.0001)

**Figure 3:**
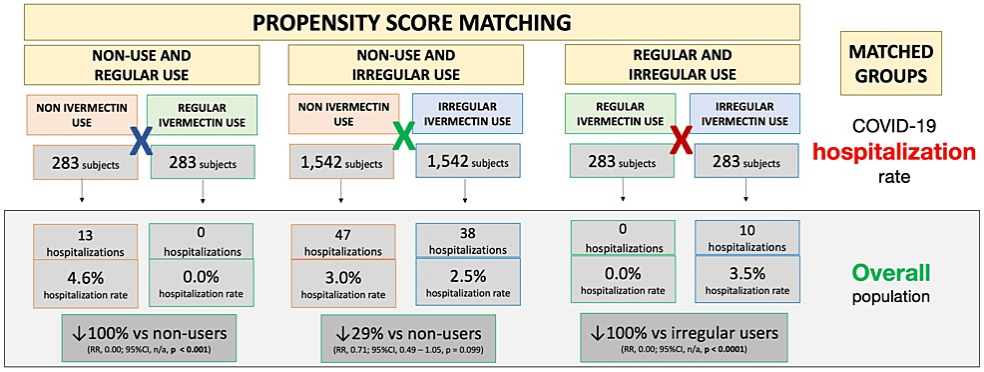
Hospitalization rates for the overall population in post-matched groups RR = risk ratio; CI = confidence interval.

Figure 3 illustrates differences in hospitalization rates in the overall population between matched groups. Balanced and matched groups of non-users and regular users (283 subjects in each group) showed 13 hospitalizations among non-users (4.6% hospitalization rate) and zero hospitalizations among regular users (0.0% hospitalization rate), a 100% reduction after adjustment for variables (RR: 0.00; 95% CI: not applicable (n/a); p < 0.0001). Between non-users and irregular users (n = 1,542 in each group), there were 47 hospitalizations among non-users (3.0% hospitalization rate) and 38 hospitalizations among irregular ivermectin users (2.5% hospitalization rate), i.e., a 29% reduction (RR: 0.71; 95% CI: 0.49 - 1.05; p = 0.099). Between regular and irregular users (n = 283 in each group), there were 10 hospitalizations among irregular users (3.5% hospitalization rate) and zero hospitalizations among regular users (0.0% hospitalization rate), i.e., a 100% reduction after adjustment for variables (RR: 0.00; 95% CI: n/a; p < 0.0001). Precise comparisons between subpopulations of regular users and non-users and between regular users and irregular users were precluded due to a lack of hospitalizations among regular users, as observed in Figure 3.

Mortality rates among ivermectin non-users, regular users, and irregular users

**Figure 4:**
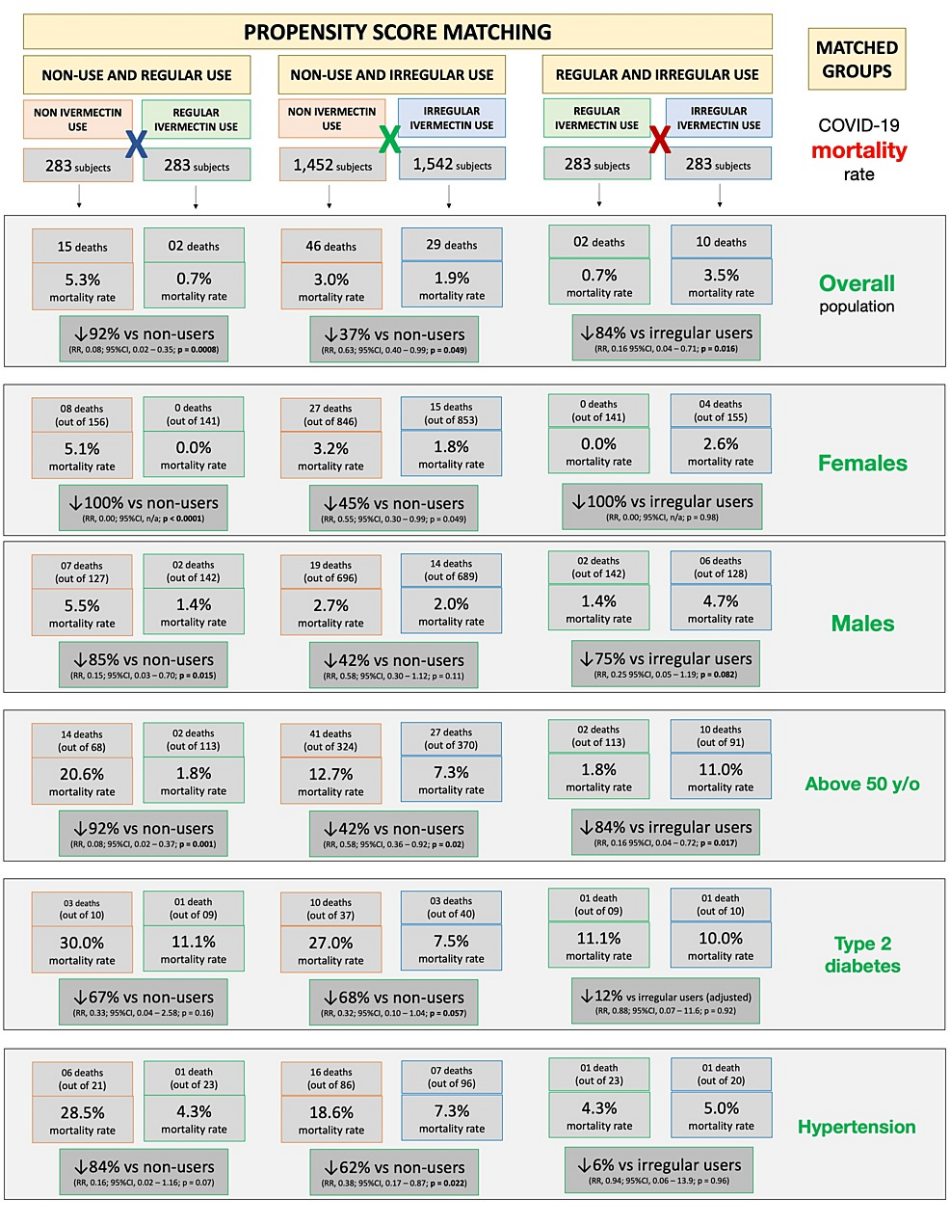
Mortality rates in the post-matched, overall population, and subpopulation groups RR = risk ratio; CI = confidence interval.

Supplement Appendix 1B shows mortality rates in ivermectin non-users, regular users, and irregular users before matching is described. Tables 6-8 and Figure 4 show mortality rates for each of the three combinations of post-matched groups of ivermectin non-users and regular users, non-users and irregular users, and regular and irregular users.

**Table 6:** Mortality rates in the three ivermectin two-group matches of non-users and regular users MI = myocardial infarction; COPD = chronic obstructive pulmonary disease; y/o = years old; CI = confidence interval; n/a = not applicable.

Propensity score matched non-users and regular users	Ivermectin non-users (n = 283)	Regular ivermectin users (n = 283)	Unadjusted mortality risk ratio (95% CI) and p-value (p)	Multivariate adjusted mortality risk ratio (95% CI) and p-value (p)
Overall	15/283 (5.3%)	2/283 (0.7%)	0.13 (0.03 – 0.56) (0.006)	0.08 (0.02 – 0.35) (0.0008)
Age				
<30 y/o	0/63 (0.0%)	0/39 (0.0%)	1.61 (0.03 – 82.7) (0.81)	n/a (1.00)
30-50 y/o	1/152 (0.7%)	0/131 (0.0%)	0.38 (0.02 – 9.51) (0.56)	n/a (0.98)
>50 y/o	14/68 (20.6%)	2/113 (1.8%)	0.07 (0.02 – 0.32) (0.0006)	0.08 (0.02 – 0.37) (0.001)
Sex				
Female	8/156 (5.1%)	0/141 (0.0%)	0.06 (0.004 – 1.08) (0.056)	0.00 (0.00 – 0.00) (<0.0001)
Male	7/127 (5.5%)	2/142 (1.4%)	0.24 (0.05 – 1.20) (0.083)	0.15 (0.03 – 0.70) (0.015)
Race				
Afro-Brazilian	1/9 (11.1%)	0/4 (0.0%)	0.63 (0.02 – 18.8) (0.79)	n/a
Mixed	1/58 (1.7%)	0/58 (0.0%)	0.33 (0.01 – 8.21) (0.50)	n/a
Caucasian	13/213 (6.1%)	2/221 (0.9%)	0.15 (0.03 – 0.66) (0.012)	n/a
Asian-Brazilian	0/3 (0.0%)	0/0	7.00 (0.05 – 953.3) (0.44)	n/a
Type 2 diabetes				
Yes	3/10 (30.0%)	1/9 (11.1%)	0.29 (0.02 – 3.48) (0.33)	0.33 (0.04 – 2.58) (0.16)
No	12/273 (4.4%)	1/274 (0.4%)	0.08 (0.01 – 0.62) (0.015)	0.05 (0.01 – 0.37) (0.004)
Hypertension				
Yes	6/21 (28.5%)	1/23 (4.3%)	0.11 (0.01 – 1.04) (0.054)	0.16 (0.02 – 1.16) (0.07)
No	9/262 (3.4%)	1/260 (0.4%)	0.11 (0.01 – 0.86) (0.036)	0.06 (0.01 – 0.49) (0.009)
Asthma				
Yes	0/0	0/0	n/a	n/a
No	15/283 (5.3%)	2/283 (0.7%)	0.13 (0.03 – 0.56) (0.006)	n/a
COPD				
Yes	0/0	0/1 (0.0%)	0.33 (0.002 – 52.6) (0.67)	n/a
No	15/283 (5.3%)	2/282 (0.7%)	0.13 (0.03 – 0.56) (0.007)	n/a
Other respiratory diseases				
Yes	0/1 (0.0%)	0/1 (0.0%)	1.00 (0.01 – 92.4) (1.00)	n/a
No	15/282 (5.3%)	2/282 (0.7%)	0.13 (0.03 – 0.56) (0.006)	n/a
Cardiovascular diseases				
Yes	0/1 (0.0%)	0/2 (0.0%)	0.60 (0.007 – 49.5) (0.82)	n/a
No	15/282 (5.3%)	2/281 (0.7%)	0.13 (0.03 – 0.56) (0.007)	n/a
Cancer				
Yes	1/2 (50.0%)	0/2 (0.0%)	0.20 (0.005 – 8.83) (0.40)	n/a
No	14/281 (5.0%)	2/281 (0.7%)	0.14 (0.03 – 0.61) (0.009)	0.09 (0.02 – 0.37) (0.001)
History of smoking				
Yes	0/2 (0.0%)	0/3 (0.0%)	0.71 (0.01 – 49.7) (0.88)	n/a (1.00)
No	15/281 (5.3%)	2/280 (0.7%)	0.13 (0.03 – 0.56) (0.007)	0.08 (0.02 – 0.36) (0.0008)
History of stroke				
Yes	1/1 (100.0%)	0/1 (0.0%)	0.11 (0.001 – 10.3) (0.34)	0.01 (0.001 – 0.02) (<0.0001)
No	14/282 (5.0%)	2/282 (0.7%)	0.14 (0.03 – 0.61) (0.009)	0.10 (0.02 – 0.40) (0.001)
History of MI				
Yes	0/0	0/0	n/a	n/a
No	15/283 (5.3%)	2/283 (0.7%)	0.13 (0.03 – 0.56) (0.007)	n/a

**Table 7:** Mortality rates in the three ivermectin two-group matches of non-users and irregular users MI = myocardial infarction; COPD = chronic obstructive pulmonary disease; y/o = years old; CI = confidence interval; n/a = not applicable.

Propensity score matched non-users and irregular users	Ivermectin non-users (n = 1,542)	Irregular ivermectin users (n = 1,542)	Unadjusted mortality risk ratio (95% CI) and p-value (p)	Multivariate adjusted mortality risk ratio (95% CI) and p-value (p)
Overall	46/1,542 (3.0%)	29/1,542 (1.9%)	0.62 (0.39 – 0.99) (0.049)	0.63 (0.40 – 0.99) (0.049)
Age				
<30 y/o	0/410 (0.0%)	0/397 (0.0%)	1.03 (0.02 – 52.2) (0.99)	1.00 (0.63 – 1.59) (1.00)
30-50 y/o	5/808 (0.6%)	2/775 (0.3%)	0.42 (0.08 – 2.15) (0.29)	0.42 (0.08 – 2.14) (0.30)
>50 y/o	41/324 (12.7%)	27/370 (7.3%)	0.54 (0.33 – 0.91) (0.019)	0.58 (0.36 – 0.92) (0.02)
Sex				
Female	27/846 (3.2%)	15/853 (1.8%)	0.54 (0.29 – 1.03) (0.061)	0.55 (0.30 – 0.99) (0.049)
Male	19/696 (2.7%)	14/689 (2.0%)	0.74 (0.37 – 1.49) (0.40)	0.58 (0.30 – 1.12) (0.11)
Race				
Afro-Brazilian	2/45 (4.4%)	0/37 (0.0%)	0.23 (0.01 – 4.99) (0.35)	n/a
Mixed	7/351 (2.0%)	7/373 (1.9%)	0.94 (0.33 – 2.71) (0.91)	0.83 (0.31 – 2.26) (0.72)
Caucasian	36/1,114 (3.2%)	21/1,102 (1.9%)	0.58 (0.34 – 1.00) (0.05)	0.53 (0.32 – 0.89) (0.016)
Asian-Brazilian	1/32 (3.1%)	1/30 (3.3%)	1.07 (0.06 – 17.9) (0.96)	0.81 (0.07 – 9.99) (0.87)
Type 2 diabetes				
Yes	10/37 (27.0%)	3/40 (7.5%)	0.22 (0.05 – 0.87) (0.031)	0.32 (0.10 – 1.04) (0.057)
No	36/1,505 (2.4%)	26/1,502 (1.7%)	0.72 (0.43 – 1.20) (0.20)	0.64 (0.39 – 1.04) (0.069)
Hypertension				
Yes	16/86 (18.6%)	7/96 (7.3%)	0.34 (0.13 – 0.88) (0.026)	0.38 (0.17 – 0.87) (0.022)
No	30/1,456 (2.1%)	22/1,446 (1.5%)	0.73 (0.42 – 1.27) (0.26)	0.66 (0.39 – 1.12) (0.12)
Asthma				
Yes	1/6 (16.7%)	1/6 (16.7%)	1.00 (0.05 – 20.8) (1.00)	3.95 (0.02 – 789.8) (0.61)
No	45/1,536 (2.9%)	28/1,536 (1.8%)	0.62 (0.38 – 0.99) (0.046)	0.56 (0.36 – 0.88) (0.011)
COPD				
Yes	0/1 (0.0%)	0/1 (50.0%)	1.00 (0.01 – 92.4) (1.00)	1.00 (0.64 – 1.56) (1.00)
No	46/1,541 (3.0%)	29/1,541 (1.9%)	0.62 (0.39 – 0.99) (0.049)	0.56 (0.36 – 0.88) (0.011)
Other respiratory diseases				
Yes	1/3 (33.3%)	0/3 (0.0%)	0.24 (0.01 – 8.62) (0.43)	0.06 (0 – 4178.4) (0.62)
No	45/1,539 (2.9%)	29/1,539 (1.9%)	0.64 (0.40 – 1.02) (0.062)	0.58 (0.37 – 0.91) (0.017)
Cardiovascular diseases				
Yes	1/9 (11.1%)	0/16 (0.0%)	0.17 (0.01 – 4.68) (0.30)	0.04 (0.03 – 0.07) (<0.0001)
No	45/1,533 (2.9%)	29/1,526 (1.9%)	0.64 (0.40 – 1.03) (0.064)	0.57 (0.36 – 0.88) (0.012)
Cancer				
Yes	1/6 (16.7%)	0/6 (0.0%)	0.28 (0.01 – 8.42) (0.47)	n/a
No	45/1,536 (2.9%)	29/1,536 (1.9%)	0.64 (0.40 – 1.02) (0.062)	0.58 (0.37 – 0.90) (0.016)
History of smoking				
Yes	1/21 (4.8%)	0/23 (0.0%)	0.29 (0.01 – 7.54) (0.46)	0.00 (<0.0001)
No	45/1,521 (3.0%)	29/1,519 (1.9%)	0.64 (0.40 – 1.02) (0.063)	0.57 (0.37 – 0.90) (0.015)
History of stroke				
Yes	0/2 (0.0%)	0/3 (0.0%)	0.71 (0.01 – 49.7) (0.88)	0.40 (0.26 – 0.62) (<0.0001)
No	46/1,540 (3.0%)	29/1,539 (1.9%)	0.62 (0.39 – 0.99) (0.049)	0.56 (0.36 – 0.88) (0.011)
History of MI				
Yes	0/1 (0.0%)	0/3 (0.0%)	0.43 (0.01 – 33.6) (0.70)	0.04 (0.03 – 0.07) (<0.0001)
No	46/1,541 (3.0%)	29/1,539 (1.9%)	0.62 (0.39 – 0.99) (0.049)	0.57 (0.36 – 0.88) (0.012)

**Table 8:** Mortality rates in the three ivermectin two-group matches of regular users and irregular users MI = myocardial infarction; COPD = chronic obstructive pulmonary disease; y/o = years old; CI = confidence interval; n/a = not applicable.

Propensity score matched regular users and irregular users	Regular ivermectin users (n = 283)	Irregular ivermectin users (n = 283)	Unadjusted mortality risk ratio (95% CI) and p-value (p)	Multivariate adjusted mortality risk ratio (95% CI) and p-value (p)
Overall	2/283 (0.7%)	10/283 (3.5%)	0.19 (0.04 – 0.89) (0.036)	0.16 (0.04 – 0.71) (0.016)
Age				
<30 y/o	0/39 (0.0%)	0/60 (0.0%)	1.53 (0.03 – 78.8) (0.83)	1.00 (0.22 – 4.46) (1.00)
30-50 y/o	0/131 (0.0%)	0/132 (0.0%)	1.01 (0.02 – 51.2) (1.00)	1.00 (0.22 – 4.46) (1.00)
>50 y/o	2/113 (1.8%)	10/91 (11.0%)	0.15 (0.03 – 0.68) (0.015)	0.16 (0.04 – 0.72) (0.017)
Sex				
Female	0/141 (0.0%)	4/155 (2.6%)	0.12 (0.01 – 2.23) (0.15)	0.00 (n/a) (0.98)
Male	2/142 (1.4%)	6/128 (4.7%)	0.29 (0.06 – 1.47) (0.13)	0.25 (0.05 – 1.19) (0.082)
Race				
Afro-Brazilian	0/4 (0.0%)	0/5 (0.0%)	1.22 (0.02 – 74.7) (0.92)	n/a
Mixed	0/58 (0.0%)	3/68 (4.4%)	0.16 (0.01 – 3.16) (0.23)	n/a
Caucasian	2/221 (0.9%)	7/209 (3.3%)	0.26 (0.05 – 1.28) (0.099)	n/a
Asian-Brazilian	0/0	0/1 (0.0%)	3.00 (0.02 – 473.1) (0.67)	n/a
Type 2 diabetes				
Yes	1/9 (11.1%)	1/10 (10.0%)	1.13 (0.06 – 21.1) (0.94)	0.88 (0.07 – 11.6) (0.92)
No	1/274 (0.4%)	9/273 (3.3%)	0.11 (0.01 – 0.85) (0.035)	0.09 (0.01 – 0.69) (0.021)
Hypertension				
Yes	1/23 (4.3%)	1/20 (5.0%)	0.86 (0.05 – 14.8) (0.92)	0.94 (0.06 – 13.9) (0.96)
No	1/260 (0.4%)	9/263 (3.4%)	0.11 (0.01 – 0.79) (0.036)	0.09 (0.01 – 0.67) (0.019)
Asthma				
Yes	0/0	0/0	n/a	n/a
No	2/283 (0.7%)	10/283 (3.5%)	0.19 (0.04 – 0.89) (0.036)	0.16 (0.04 – 0.71) (0.016)
COPD				
Yes	0/1 (0.0%)	0/0	0.33 (0.002 – 52.6) (0.67)	n/a
No	2/282 (0.7%)	10/283 (3.5%)	0.19 (0.04 – 0.90) (0.036)	0.16 (0.04 – 0.72) (0.017)
Other respiratory diseases				
Yes	0/1 (0.0%)	0/0	n/a	n/a
No	2/282 (0.7%)	10/283 (3.5%)	0.19 (0.04 – 0.90) (0.036)	0.16 (0.04 – 0.72) (0.017)
Cardiovascular diseases				
Yes	0/2 (0.0%)	0/5 (0.0%)	2.20 (0.03 – 146.1) (0.71)	0.52 (0.11 – 2.30) (0.38)
No	2/281 (0.7%)	10/278 (3.6%)	0.19 (0.04 – 0.88) (0.034)	0.16 (0.04 – 0.71) (0.016)
Cancer				
Yes	0/2 (0.0%)	0/2 (0.0%)	1.00 (0.01 – 73.3) (1.00)	1.00 (0.22 – 4.46) (1.00)
No	2/281 (0.7%)	10/281 (3.6%)	0.19 (0.04 – 0.89) (0.036)	0.16 (0.04 – 0.70) (0.016)
History of smoking				
Yes	0/3 (0.0%)	0/1 (0.0%)	0.43 (0.01 – 33.6) (0.70)	n/a
No	2/280 (0.7%)	10/282 (3.5%)	0.20 (0.04 – 0.90) (0.036)	0.16 (0.03 – 0.72) (0.017)
History of stroke				
Yes	0/1 (0.0%)	0/1 (0.0%)	1.00 (0.01 – 92.4) (1.00)	1.00 (0.22 – 4.46) (1.00)
No	2/282 (0.7%)	10/282 (3.5%)	0.19 (0.04 – 0.89) (0.036)	0.16 (0.04 – 0.72) (0.017)
History of MI				
Yes	0/0	0/0	n/a	n/a
No	2/283 (0.7%)	10/283 (3.5%)	0.19 (0.04 – 0.89) (0.036)	0.16 (0.04 – 0.71) (0.016)

Between matched groups of non-users and regular users (n = 283 in each group), mortality rate was 5.3% (15 deaths) among non-users and 0.7% (two deaths) among regular users; a 92% reduction in mortality rate (RR: 0.08; 95% CI: 0.02 - 0.35; p = 0.00083). Compared to non-users, reductions in mortality rate among regular users were 100% among females (eight deaths among 156 non-users and zero deaths among 141 regular users; RR: 0.00; 95% CI: n/a; p < 0.0001) and 85% among males (seven deaths among 127 non-users and two deaths among 142 regular users; RR: 0.15; 95% CI: 0.03 - 0.70; p = 0.015) with 92% for subjects above 50 years of age (14 deaths among 68 non-users and two deaths among 113 regular users; RR: 0.08; 95% CI: 0.02 - 0.37; p = 0.001). There was statistically a non-significant 67% reduction for T2D (three deaths among 10 non-users and one death among nine regular users; RR: 0.33; 95% CI: 0.04 - 2.58; p = 0.16), and 84% among subjects with hypertension (six deaths among 21 non-users and one death among 23 regular users; RR: 0.16; 95% CI: 0.02 - 1.16; p = 0.07).

Between matched groups of non-users and irregular users (n = 1,542 in each group), there was a 3.0% mortality rate (46 deaths) among non-users and a 1.9% mortality rate (29 deaths) among irregular users, showing a 37% reduction in mortality rate (RR compared to non-users: 0.63; 95% CI: 0.40 - 0.99; p = 0.049). A 45% reduction in mortality rate occurred among females; 3.2% for non-users (27 death among 846) and 1.8% for irregular users (15 deaths among 853) (RR: 0.55; 95% CI: 0.30 - 0.99; p = 0.049); and 42% reduction occurred for males; 2.7% of non-users (19 deaths among 696) and 2.0% of irregular users (14 deaths among 689) (RR: 0.58; 95% CI: 0.30 - 1.12; p = 0.11). Mortality rate for subjects above 50 years old was 12.7% for 324 non-users (41 deaths) and 7.3% for 370 regular users (27 deaths); a 42% reduction in mortality rate (RR: 0.58; 95% CI: 0.36 - 0.92; p = 0.02). Participants with T2D had 27.0% mortality rate for 37 non-users (10 deaths) and 7.5% for 40 irregular users (three deaths); a 68% reduction in mortality rate among participants with T2D (RR: 0.32; 95% CI: 0.10 - 1.04; p = 0.057). Those with hypertension had a 62% reduction in mortality rate; 18.6% of 86 non-users (16 deaths) and 7.3% of 96 irregular users (seven deaths) (RR: 0.38; 95% CI: 0.17 - 0.87; p = 0.022). In sub-populations without comorbidities, reductions in mortality rates were between 40% and 45%.

When groups of regular users and irregular users are matched (283 subjects in each group), there was a 0.7% and 3.5% (two deaths and 10 deaths) mortality rate among regular and irregular users, reflecting a multivariate-adjusted 84% reduction in mortality rate (RR: 0.16; 95% CI: 0.04 - 0.71; p = 0.016). The small number of events between these two groups precludes more statistically significant differences, despite large effect size and differences, in particular in subgroups with fewer subjects. The mortality rate was 2.6% (four deaths out of 155) among non-user females and 0.0% (out of 141 females) among regular user females. There was a 4.7% mortality rate (six deaths) among 128 non-user males and a 1.4% mortality rate (two deaths) among 142 regular user males, showing a reduction of 75% (RR: 0.25; 95% CI: 0.05 - 1.19; p = 0.082) in mortality rate. Reduction in mortality rate was 84% for those over 50 years of age; 11.0% for non-users (10 deaths among 91) and 1.8% for regular users (two deaths among 113) (RR: 0.16; 95% CI: 0.04 - 0.72; p = 0.017). Among participants with T2D, mortality rate was 10.0% (one death) among 10 irregular users and 11.1% (one death) among nine regular users, which is statistically similar between groups (RR: 0.88; 95% CI: 0.07 - 11.6; p = 0.92). Subjects with hypertension had 5.0% mortality rate (one death) among 20 irregular users and 4.3% mortality rate (one death) among 23 regular users, which is similar between groups (RR: 0.94; 95% CI: 0.06 - 13.9; p = 0.96).

Risk of dying from COVID-19 between ivermectin non-users, regular users, and irregular users

**Figure 5:**
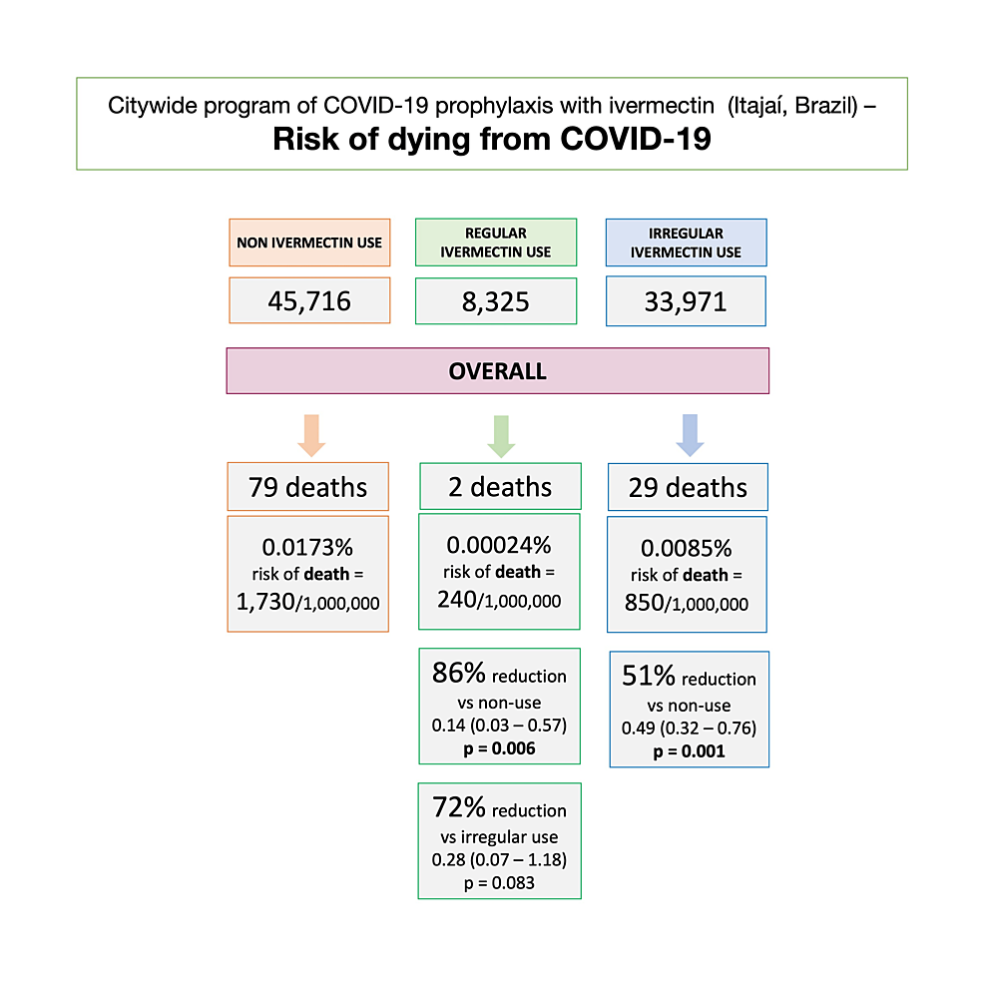
Risk of dying from COVID-19 among ivermectin non-users, regular users, and irregular users RR = risk ratio; CI = confidence interval.

Considering the population and participants of Itajaí, as well as inhabitants of Itajaí, who did not use ivermectin prophylactically, the unadjusted risk of dying from COVID-19 was 1,730 in every 1,000,000 subjects among non-users, 240 among regular users, and 850 among irregular users. Compared to non-users, the risk of dying from COVID-19 was 86% lower in regular users (RR: 0.14; 95% CI: 0.03 - 0.57; p = 0.006) and 51% lower in irregular users (RR: 0.49; 95% CI: 0.32 - 0.76; p = 0.001). The risk of dying from COVID-19 was 72% lower in regular users than irregular users (RR: 0.28; 95% CI: 0.07 - 1.18; p = 0.089). Figure 5 illustrates the risk of dying from COVID-19 in each population.

## Discussion

The program in Itajaí, Brazil: ivermectin prophylaxis for COVID-19

The present study provides in-depth results on the prospective study of ivermectin as prophylaxis for COVID-19 in Itajaí, located in Southern Brazil. Particularities of Itajaí included its dynamic population due to the presence of an overwhelmingly large port compared to the size of the city. This explained why the city was one of the first in the state to reach 1,000 cases in 2020 [26]. In the past, the city experienced some of the highest rates of HIV infections in Brazil [27], partially substantiated by being a port city, an "independent" predictor of a higher prevalence of HIV infection [28].

The decision to adopt a prophylaxis program with ivermectin in Itajaí was based on (1) the fact that case numbers rose rapidly and at a higher speed than in other cities; (2) the inability to isolate port workers in the absence of pharmacological or non-pharmacological therapies for COVID-19; (3) because it had already been proven to be a potent antiviral for over 20 viruses, studied independently and peer-reviewed, including the first severe acute respiratory syndrome coronavirus (SARS-CoV) epidemic before the COVID-19 pandemic; and (4) the extensive safety profile and favorable cost-effectiveness of ivermectin. Hence, the program of Itajaí strictly followed all bioethical principles using ivermectin as prophylaxis for COVID-19. The ivermectin was offered optionally, as prophylaxis for COVID-19, following medical screening by medical doctors.

Ivermectin as a defense against all major COVID-19 outcomes: does it depend on the regularity of ivermectin use?

In our first paper [25], ivermectin was shown to be associated with significant reductions in infection rate (44%), hospitalization rate (56%), and mortality rate (68%), when compared to subjects that did not use ivermectin prophylactically and irrespective of the regularity of ivermectin use.

**Figure 6:**
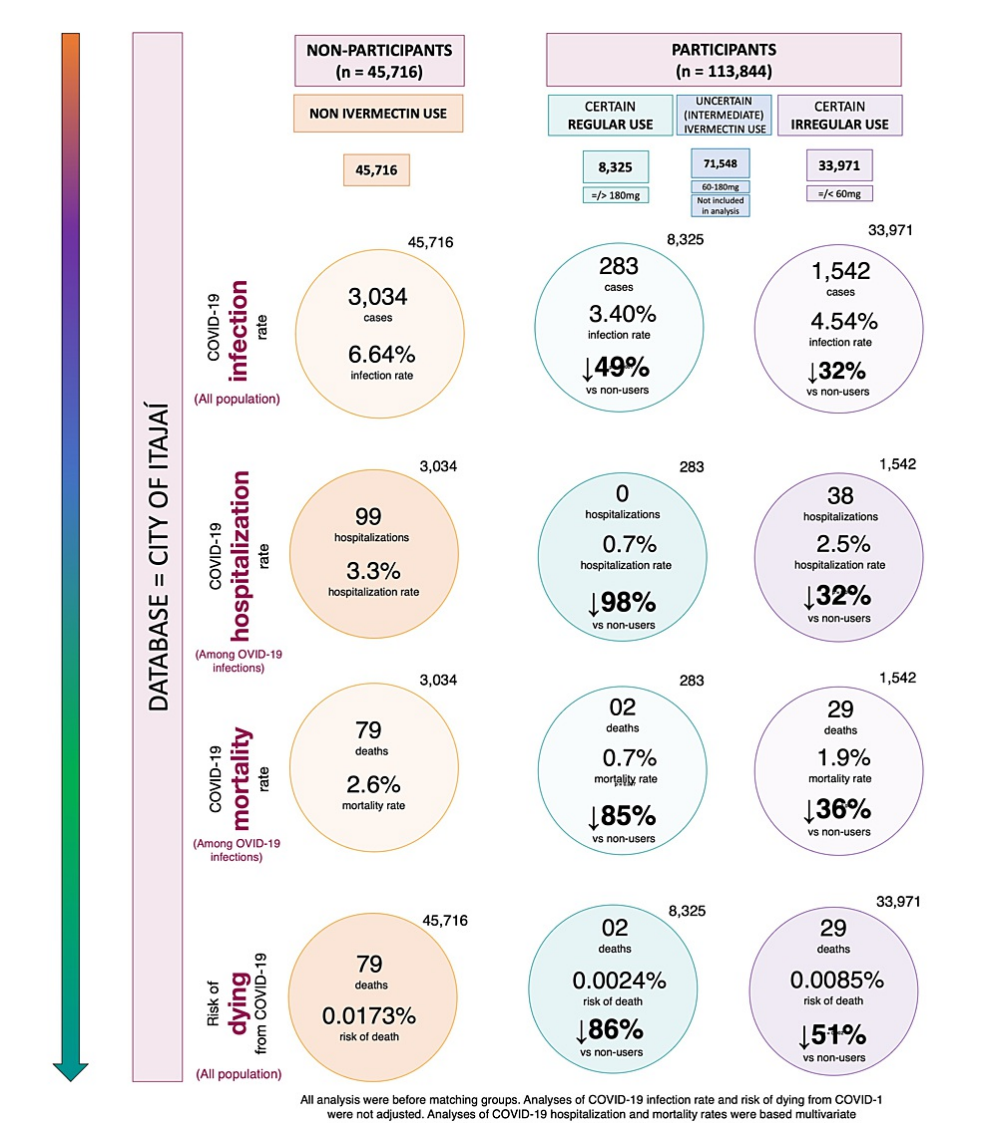
COVID-19 infection, hospitalization, mortality rates, and risk of dying from COVID-19 across different patterns of ivermectin use

This study paper analyzes the impact of the regular use of ivermectin on COVID-19 infection. This impact included non-users, regular users, and irregular ivermectin users. These groups were estimated from the matched population in the city of Itajaí, with an impressive 100% of the population of Itajaí being digitalized in the government data system. Their COVID-19 cases, hospitalizations in public hospitals, and all deaths due to COVID-19 were strictly followed and recorded. Figure 6 summarizes an overall view of the findings of this study.

This reduction of COVID-19 infection had a significant effect on the reduction of transmission and perpetuation of the pandemic in Itajaí. Also, the reduction of related hospitalizations and mortality is indisputably meaningful. They reduced not only costs and pressure on the health system but saved many lives.

Ivermectin regular users were older (average age = 47 years) compared to irregular users (average age = 41 years). The non-users (average age = 39.8 years) had approximately 20% to 50% higher prevalence of T2D and hypertension. If ivermectin did not work, one would expect higher hospitalizations and mortality rates in the group of regular users, which did not happen, as seen in the pre-matched analysis, in Supplement Appendix 1.

Notably, there were no hospitalizations for any of the 289 regular users. After observing matching between groups, the reduction in hospitalization rate was 100% in regular users compared to non-users and irregular users. Analysis of sub-populations in these two comparisons was unfeasible due to the lack of hospitalizations for the regular users. Statistically significant reductions were observed in the hospitalization rate for irregular users when compared to non-users (35% reduction; p = 0.03), which was more relevant in high-risk populations. This included subjects 50 years of age and above (reduction of 38%; p = 0.027) and those with comorbidities. A 69% reduction was seen among subjects with T2D (p = 0.063), 45% among subjects with hypertension (p = 0.10), and 73% among subjects with cardiovascular diseases (p = 0.23), with reductions similar between males and females. This means that even with uncontrolled, irregular use of ivermectin, there is a significant reduction in the number of hospitalizations in COVID-19-infected participants.

The regularity of ivermectin intake demonstrated a progressive impact on the reduction of mortality rate, which was more clearly observed after matching groups. The regular users showed a 90% mortality rate reduction compared to non-users (p = 0.003) and 79% reduction compared to irregular users (p = 0.05). Irregular users had a reduction of 37% compared to non-users (p = 0.63). Reductions among regular users were similar (between 86% and 89%) across different high-risk populations (50 years old and above with comorbidities). High-risk populations of irregular users had reductions in mortality rate between 34% and 60% compared to non-users. The most profoundly significant results were for women who used ivermectin regularly, with no deaths among all 144 participants.

The risk of dying from COVID-19, when considering the whole population, was notably lower among regular users, compared to both non-users (86% reduction) and irregular users (72% reduction). This risk was also lower among irregular users compared to non-users (51% reduction). Since baseline characteristics were not present for non-user, non-infected subjects, there were no adjustments to be done for variables relative to their chances of dying from COVID-19.

In common, all outcomes related to COVID-19 infection demonstrated a dose-related response effect, with greater reductions in all outcomes with the higher ivermectin intake. This strong correlation reinforces the causal relationship between ivermectin intake and protection from COVID-19. Also, although regular users still had COVID-19 cases (with a lower infection rate than non-users), these cases tended to be milder, compared to non-users or irregular users, as observed in the significant absence of hospitalizations and deaths.

Mechanistically, the accumulated dose of ivermectin, consequently obtained with the regular use of ivermectin, had a strong impact on COVID-19-related outcomes, i.e., once infected, higher amounts of ivermectin administered related to a better prognosis. Of note, the strict control of which days ivermectin was used did not affect the results.

Although a demonstrative dose-response was observed consistently across the groups (non-users, regular users, and irregular users) unexpectedly, the risk of COVID-19 infection was not largely influenced by the regularity of ivermectin use (Figure 2). The possible long-term actions of ivermectin, that go beyond its serum or cytoplasmatic concentration, may explain the progressive protection with a higher regularity of ivermectin use.

Our results demonstrated protection against COVID-19 when regularly used for two days, every 15 days regimen. This prophylactic treatment regimen respected the already extensively known safety profile of ivermectin, since, notably, it did not surpass the usual doses for scabies.

Noteworthy aspects of the study

Regularity is defined as something happening repeatedly in a fixed pattern. As such, this study determined the criteria for regularity to be more than 30 tablets of ivermectin over five months, with a continuous supply of ivermectin, determined by the number of tablets prescribed and taken every other week over 12 weeks.

To determine different outcomes, it was critical that a correct baseline population was established for each outcome. Because there were more than 8,000 subjects from outside the city of Itajaí that participated in the study, the infection rate could not be calculated based on the participating subjects because COVID-19 cases from other cities were underreported in Itajaí among ivermectin non-users. In fact, the “infection rate” of overall participants (1.40%) among subjects from other cities (177 cases out of 8,352 subjects) was much lower than the infection rates within the city of Itajaí. This clearly demonstrated underreporting. Calculations were based on participants from Itajaí only, for which COVID-19 cases were strictly controlled. Correspondingly, the risk of dying from COVID-19 aims to evaluate the risk of an undesired outcome irrespective of how many cases occurred, unlike the mortality rate that included the full population.

The use of ivermectin was able to reduce COVID-19 infection significantly. A small portion of regular users was sufficient to positively affect the city’s numbers related to COVID-19. Unfortunately, because most of the population failed to continue in a program of prophylactic ivermectin use, the rise in cases after July 7, 2020, in the state of Santa Catarina, led to a skewed perception to potentially discredit the efficacy of ivermectin. However, misleading this perception, a committed program of ivermectin could have led to a huge positive health impact across the whole state.

Unexpectedly, the different regularity of ivermectin use did not show significant changes in the reduction of COVID-19 infections. One could speculate that subjects that did not obtain ivermectin from the program in a regular manner may have acquired ivermectin over the counter, where it was available. However, during the first two months of the program, Brazil experienced not only a temporary shortage of ivermectin due to a sudden increase in demand, but required a medical prescription and experienced an associated price increase by five times, precluding its use outside the program. More importantly, while infection rates did not reduce with regular use of ivermectin, compared to irregular users, hospitalization and mortality rates reduced substantially, showing a dose-effect response of ivermectin for COVID-19-related outcomes.

The apparent contradictory lack of hospitalizations, while there were two deaths in the group of regular users, may be explained by the fact that patients either used a private hospital outside the city of Itajaí or in an institution that was not a hospital. Death reports are mandatorily for public and private hospitals; however, hospitalizations are not reported. Another hypothesis is that these deaths occurred without hospitalization. Depending on the characteristics and social context of these participants, this is not unusual when hospitals get overwhelmed, or when patients avoid seeking hospital care for other reasons [29].

Limitations

Updated medical histories were done for ivermectin users at follow-up appointments with medical doctors from the SUS. Regarding the non-users, the participants did not have follow-ups to update their medical records. Depending on the calculation methods performed for infection rates, this could create some differences. Imprecisions and modifications evident, although minimal, between the first manuscript [25] and this study, did not impact the fact that ivermectin use reduced COVID-19-related outcomes. In addition, in the present analysis, we did not control for the COVID-19 infection dates. Of note, although there were no other hospitals in Itajaí, due to the limited capacity of the city hospital, some patients with health insurance were transferred to private hospitals outside of Itajaí, while some patients without private insurance were cared for in institutions that were not hospitals. Unlike hospitalizations, deaths were mandatorily reported, which precluded any imprecision in the calculations of the mortality rate.

The number of tablets was calculated according to body weight. Most of the population used between two and three tablets daily for two days, every 15 days. Due to the minimal difference between the number of ivermectin tablets used, the amount used (frequency of its use) could be determined with a reasonable level of precision.

This observational study obtained results that presented a high level of certainty by employing strict control of the data outcome among COVID-19 cases and strict control of the number of deaths due to COVID-19 in the overall population. The fact that PSM was employed for outcomes in such a large population makes these data reliable, being sourced from official government databases (datasets: https://osf.io/uxhaf/).

Final discussion

Regular use of ivermectin led to a 100% reduction in hospitalization rate, a 92% reduction in mortality rate, and an 86% reduction in the risk of dying from COVID-19 when compared to non-users. Irregular use of ivermectin led to a 51% reduction in the risk of dying, a 29% reduction in hospitalization rate, and a 37% reduction in mortality rate from COVID-19. Statistically significant reductions in hospitalization (100%) and mortality rates (84%), and risk of dying from COVID-19 (72%) were observed in regular users when compared to irregular users. The response pattern of ivermectin use and level of protection from COVID-19-related outcomes was identified and consistent across dose-related levels. The reduction in COVID-19 infection rate occurred in a consistent and significant dose-dependent manner, with reductions of 49% and 32% in regular and irregular users, when compared to non-users. The most striking evidence of ivermectin's effectiveness was the 100% reduction in mortality for female regular users.

The analysis of the data gathered from official government databases showed that ivermectin had an impactful reduction in the incidence of COVID-19 infection, in a dose-response manner. Even for irregular users, benefits were observed.

The data conclusively show that the risk of dying from COVID-19 was lower for all regular and irregular users of ivermectin, compared to non-users, considering the whole population.

A progressive, dose-response pattern of protection from COVID-19-related outcomes was observed and consistent across all levels of ivermectin used. Consequently, the findings in this study show how the risk of contracting COVID-19 infection was not greatly influenced by the regularity (regular user = 3.4%; irregular user = 4.54%) of ivermectin use, making it very significant as a preventive therapy for COVID-19.

Finally, the evidence, in this study, added to the efficacy of ivermectin as prophylaxis for COVID-19. There are no equivalent RCTs when it comes to the effects of prophylaxis since this was an observational study of a strictly controlled population with a great level of control for confounding factors at a magnitude unfeasible to be conducted in an RCT. This study demonstrated the effects of ivermectin in real life in an overwhelmingly precise manner, close to post-RCT real-life studies [30-32]. The evidence provided by the present study is among the strongest and most conclusive data regarding ivermectin efficacy.

## Conclusions

The regular use of ivermectin decreased hospitalization for COVID-19 by 100%, mortality by 92%, and the risk of dying from COVID-19 by 86% when compared to non-users.

Protection from COVID-19-related outcomes was observed across all levels of ivermectin use, with a notable reduction in risk of death in the over 50-year-old population and those with comorbidities. The reduction in infection rate was significant, irrespective of the level of ivermectin use. The results of this prospective observational study of a strictly controlled population of 223,128 participants reinforce the efficacy of ivermectin and the demonstration of a dose-response effect.

## Figures and Tables

**Figure 7:**
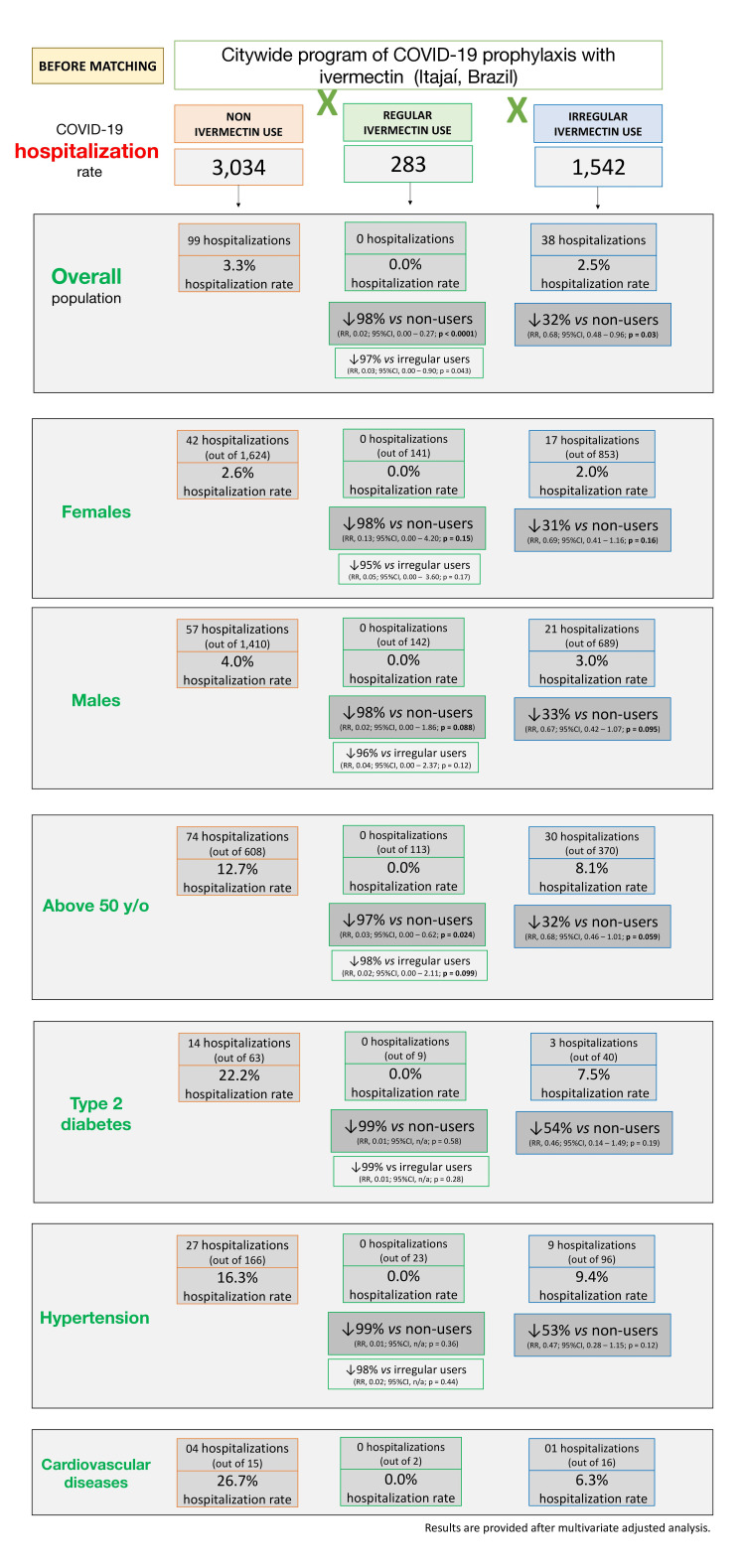
Hospitalization rates before matching in ivermectin non-users, irregular users, and regular users RR = risk ratio; CI = confidence interval.

**Figure 8:**
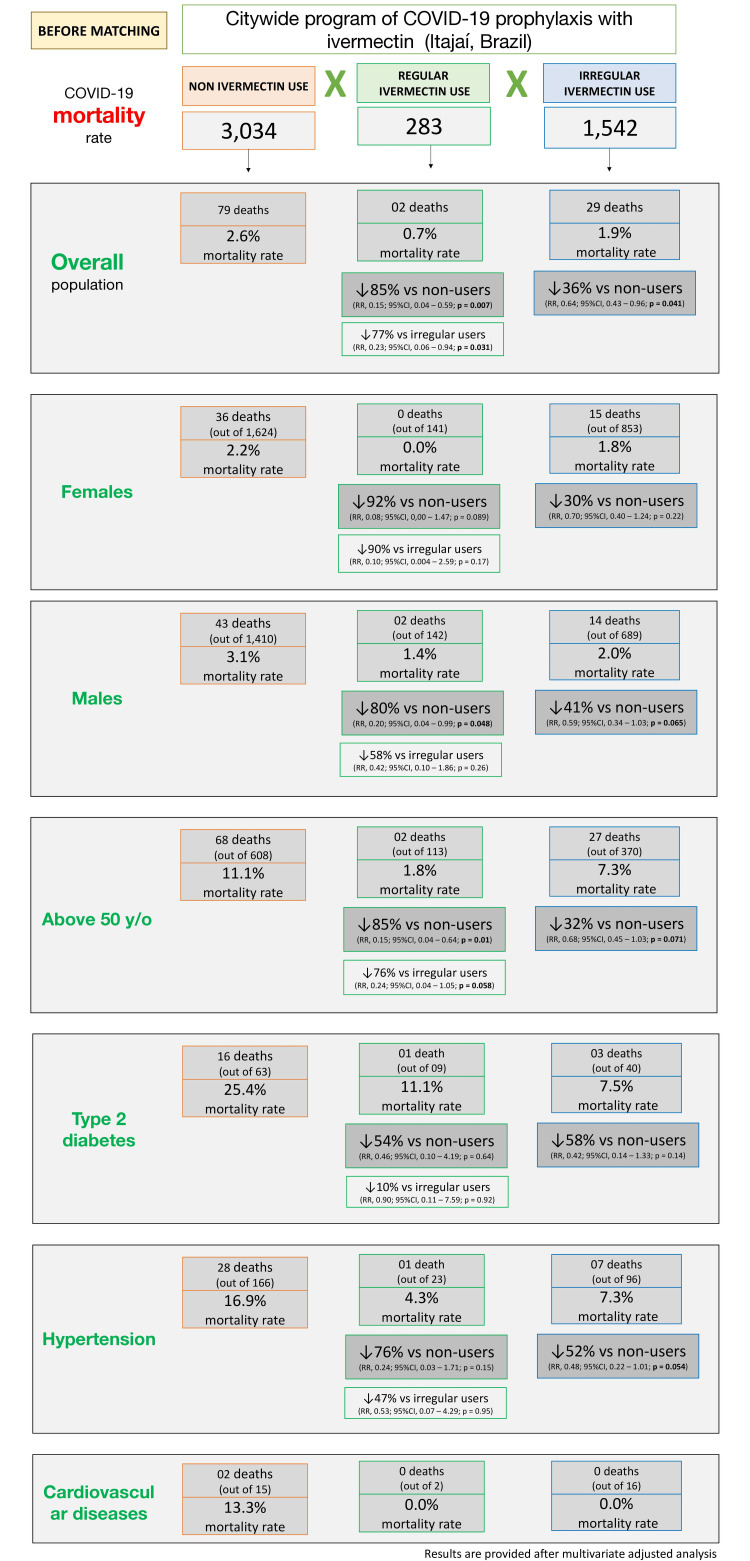
Pre-matching mortality rates in ivermectin non-users, irregular users, and regular users RR = risk ratio; CI = confidence interval.

## Appendices

Supplement appendix 1

Regular use of ivermectin as prophylaxis for COVID-19 led up to a 92% reduction in COVID-19 mortality rate in a dose-response manner: results of a prospective observational study of a strictly controlled population of 88,012 subjects.

Index

Appendix 1A: Hospitalization rates in non-users, irregular users, and regular users before matching

Appendix 1B: Mortality rates in non-users, irregular users, and regular users before matching

Appendix 1A: hospitalization rates in non-users, irregular users, and regular users before matching

Tables 9, 10 describe hospitalization rates in ivermectin non-users, irregular users, and strictly regular users, before matching groups, and head-to-head comparisons between groups, and Figure 7 illustrates the findings in overall and relevant subpopulations. Before matching, there were no hospitalizations among ivermectin regular users, 38 hospitalizations among irregular users (2.5%), and 99 hospitalizations among non-users (3.3%). After adjustment for variables, reduction in hospitalization rate in ivermectin regular users was 98% compared to non-users (RR: 0.02; 95% CI: 0.00 - 0.27; p < 0.0001) and 97% compared to irregular users (RR: 0.03; 95% CI: 0.00 - 0.90; p < 0.043).

**Table 9:** Pre-matching hospitalization rates in overall and in subpopulations among ivermectin non-users, irregular users, and regular users MI = myocardial infarction; COPD = chronic obstructive pulmonary disease; y/o = years old; CI = confidence interval; n/a = not applicable.

Hospitalization rates	Non-matched (pre-matching) groups				Overall p-values	
Population	Overall (n = 4,859)	Non-users - hospitalizations (%) (n = 3,034)	Regular users - hospitalizations (%) (n = 283)	Irregular users - hospitalizations (%) (n = 1,542)	Unadjusted overall p-value	Unadjusted overall p-value (p) and risk ratio (95% CI) between subpopulations
Overall	137/4,859 (2.8%)	99/3,034 (3.3%)	0/283 (0.0%)	38/1,542 (2.5%)	<0.0001	
Age						<0.0001
<30 y/o	2/1,280 (0.2%)	1/844 (0.1%)	0/39 (0.0%)	1/397 (0.3%)	0.46	
30-50 y/o	12/2,488 (1.2%)	24/1582 (1.5%)	0/131 (0.0%)	7/775 (0.9%)	<0.0001	3.10 (0.69 – 13.9) (0.14) vs <30 y/o
>50 y/o	104/1,091 (9.5%)	74/608 (12.7%)	0/113 (0.0%)	30/370 (8.1%)	<0.0001	67.3 (16.6 – 273.5) (<0.0001) vs <30 y/o / 21.7 (11.9 – 39.7) (<0.0001) vs 30-50 y/o
Sex						1.36 (0.95 – 1.93) (0.09) vs female
Female	59/2,618 (2.3%)	42/1,624 (2.6%)	0/141 (0.0%)	17/853 (2.0%)	0.004	
Male	68/2,241 (3.0%)	57/1,410 (4.0%)	0/142 (0.0%)	21/689 (3.0%)	<0.0001	
Race						0.79
Afro-Brazilian	4/141 (2.8%)	4/100 (4.0%)	0/4 (0.0%)	0/37 (0.0%)	n/a	0.98 (0.35 – 2.69) (0.96) vs Caucasians / 1.05 (0.37 – 3.04) (0.92) vs mixed / 2.60 (0.29 – 23.6) (0.40) vs Asian-Brazilian
Mixed	30/1,113 (2.7%)	19/682 (2.8%)	0/58 (0.0%)	11/373 (2.9%)	0.14	0.93 (0.61 – 1.40) (0.72) vs Caucasians / 2.47 (0.33 – 18.3) (0.38) vs Asian-Brazilian
Caucasian	102/3,515 (2.9%)	76/2,192 (3.5%)	0/221 (0.0%)	26/1,102 (2.4%)	<0.0001	2.66 (0.37 – 19.3) (0.33) vs Asian-Brazilian
Asian-Brazilian	1/90 (1.1%)	0/60 (0.0%)	0/0 (0.0%)	1/30 (3.3%)	n/a	
Type 2 diabetes						6.90 (3.99 – 11.9) (<0.0001)
Yes	17/112 (15.2%)	14/63 (22.2%)	0/9 (0.0%)	3/40 (7.5%)	0.006	
No	120/4,747 (2.5%)	85/2,971 (2.9%)	0/274 (0.0%)	35/1,502 (2.3%)	<0.0001	
Hypertension						6.40 (4.29 – 9.56) (<0.0001)
Yes	36/285 (12.6%)	27/166 (16.3%)	0/23 (0.0%)	9/96 (9.4%)	0.0003	
No	101/4,574 (2.2%)	72/2,868 (2.5%)	0/260 (0.0%)	29/1,446 (2.0%)	<0.0001	
Asthma						3.14 (0.40 – 24.5) (0.27)
Yes	1/12 (8.3%)	0/6 (0.0%)	0/0 (0.0%)	1/6 (16.7%)	n/a	
No	136/4,837 (2.8%)	99/3,028 (3.3%)	0/283 (0.0%)	37/1,536 (2.4%)	<0.0001	
COPD						4.95 (0.61 – 40.5) (0.14)
Yes	1/8 (12.5%)	1/6 (15.7%)	0/1 (0.0%)	0/1 (0.0%)	n/a	
No	136/4,851 (2.8%)	98/3,028 (3.2%)	0/282 (0.0%)	38/1,541 (2.5%)	<0.0001	
Other respiratory diseases						4.33 (0.54 – 34.9) (0.17)
Yes	1/9 (11.1%)	1/5 (20.0%)	0/1 (0.0%)	0/3 (0.0%)	n/a	
No	136/4,850 (2.8%)	98/3,029 (3.2%)	0/282 (0.0%)	38/1,539 (2.5%)	<0.0001	
Cardiovascular diseases						6.36 (2.42 – 16.7) (0.0002)
Yes	5/33 (15.2%)	4/15 (26.7%)	0/2 (0.0%)	1/16 (6.3%)	0.058	
No	132/4,836 (2.7%)	95/3.019 (31.4%)	0/281 (0.0%)	37/1,526 (24.2%)	<0.0001	
Cancer						1.82 (0.24 – 13.7) (0.56)
Yes	1/20 (5.0%)	1/12 (8.3%)	0/2 (0.0%)	0/6 (0.0%)	n/a	
No	136/4,839 (2.8%)	98/3,022 (3.2%)	0/281 (0.0%)	38/1,536 (2.5%)	<0.0001	
Smoking						0.47 (0.07 – 3.44) (0.46)
Yes	1/73 (1.4%)	1/47 (2.1%)	0/3 (0.0%)	0/23 (0.0%)	n/a	
No	136/4,786 (2.8%)	98/2,987 (3.3%)	0/280 (0.0%)	38/1,519 (%)	<0.0001	
History of stroke						9.56 (2.64 – 34.8) (0.0006)
Yes	3/14 (21.4%)	3/10 (30.0%)	0/1 (0.0%)	0/3 (0.0%)	n/a	
No	134/4,845 (2.8%)	96/3,024 (3.2%)	0/282 (0.0%)	38/1,539 (2.5%)	<0.0001	
History of MI						26.4 (5.85 – 119.2) (<0.0001)
Yes	3/7 (42.9%)	2/4 (50.0%)	0/0 (0.0%)	1/3 (33.3%)	0.087	
No	134/4,852 (2.8%)	97/3,030 (3.2%)	0/283 (0.0%)	37/1,539 (2.4%)	<0.0001	

**Table 10:** Head-to-head comparisons of hospitalization rates between ivermectin non-users and regular users, non-users and irregular users, and irregular and regular users MI = myocardial infarction; COPD = chronic obstructive pulmonary disease; y/o = years old; CI = confidence interval; n/a = not applicable.

Hospitalization rates	Non-users versus regular users		Non-users versus irregular users		Regular versus irregular users	
Population	Unadjusted hospital risk ratio (95% CI) and p-value (p)	Multivariate adjusted hospital risk ratio (95% CI) and p-value (p)	Unadjusted hospital risk ratio (95% CI) and p-value (p)	Multivariate adjusted hospital risk ratio (95% CI) and p-value (p)	Unadjusted hospital risk ratio (95% CI) and p-value (p)	Multivariate adjusted hospital risk ratio (95% CI) and p-value (p)
Overall	0.05 (0.003 – 0.84) (0.037­)	0.02 (0.00 – 0.27) (< 0.0001)	0.75 (0.51 – 1.09) (0.14)	0.68 (0.48 – 0.96) (0.03)	0.07 (0.004 – 1.13) (0.06)	0.03 (0.00 – 0.90) (0.043)
Age						
<30 y/o	7.12 (0.29 – 177.5) (0.23)	0.53 (0.00 – 844.1) (0.87)	2.13 (0.13 – 34.1) (0.59)	1.73 (0.14 – 21.2) (0.67)	3.35 (0.13 – 83.5) (0.46)	0.35 (0.00 – 3625.6) (0.82)
30-50 y/o	0.24 (0.01 – 4.00) (0.32)	0.17 (0.01 – 5.00) (0.30)	0.59 (0.25 – 1.38) (0.22)	0.58 (0.25 – 1.33) (0.20)	0.40 (0.02 – 7.03) (0.53)	0.14 (0.001 – 14.3) (0.40)
>50 y/o	0.03 (0.002 – 0.51) (0.015)	0.03 (0.001 – 0.62) (0.024)	0.64 (0.41 – 0.99) (0.047)	0.68 (0.46 – 1.01) (0.059)	0.05 (0.003 – 0.81) (0.035)	0.02 (0.00 – 2.11) (0.099)
Sex						
Female	0.13 (0.008 – 2.15) (0.15)	0.02 (0.00 – 4.20) (0.15)	0.77 (0.43 – 1.36) (0.36)	0.69 (0.41 – 1.16) (0.16)	0.17 (0.01 – 2.82) (0.22)	0.05 (0.00 – 3.60) (0.17)
Male	0.08 (0.01 – 1.34) (0.08)	0.02 (0.00 – 1.86) (0.088)	0.75 (0.45 – 1.24) (0.26)	0.67 (0.42 – 1.07) (0.095)	0.11 (0.007 – 1.81) (0.12)	0.04 (0.00 – 2.37) (0.12)
Race						
Afro-Brazilian	2.38 (0.11 – 51.4) (0.58)	n/a	0.29 (0.01 – 5.44) (0.40)	0.04 (0.00 – 32.4) (0.35)	8.33 (0.15 – 473.6) (0.30)	n/a
Mixed	0.29 (0.02 – 4.88) (0.39)	n/a	1.06 (0.50 – 2.25) (0.88)	0.81 (0.40 – 0.98) (0.038)	0.27 (0.02 – 4.63) (0.37)	n/a
Caucasian	0.06 (0.004 – 1.01) (0.051)	n/a	0.67 (0.43 – 1.06) (0.085)	0.64 (0.43 – 0.98) (0.038)	0.09 (0.01 – 1.51) (0.095)	n/a
Asian-Brazilian	n/a	n/a	6.15 (0.24 – 155.7) (0.27)	n/a (0.42)	n/a	n/a
Type 2 diabetes						
Yes	0.18 (0.01 – 3.28) (0.25)	0.01 (n/a) (0.58)	0.28 (0.08 – 1.06) (0.061)	0.46 (0.14 – 1.49) (0.19)	0.56 (0.03 – 11.9) (0.71)	0.01 (n/a) (0.62)
No	0.06 (0.004 – 0.99) (0.049)	0.04 (0.002 – 0.54) (0.016)	0.34 (0.11 – 1.04) (0.059)	0.71 (0.49 – 1.03) (0.067)	0.08 (0.005 – 1.24) (0.07)	0.05 (0.002 – 0.96) (0.047)
Hypertension						
Yes	0.11 (0.01 – 1.83) (0.12)	0.01 (n/a) (0.36)	0.53 (0.24 – 1.19) (0.12)	0.57 (0.28 – 1.15) (0.12)	0.20 (0.01 – 3.49) (0.27)	0.02 (n/a) (0.44)
No	0.07 (0.005 – 1.20) (0.067)	0.04 (0.002 – 0.66) (0.025)	0.79 (0.51 – 1.23) (0.30)	0.72 (0.48 – 1.09) (0.12)	0.09 (0.01 – 1.51) (0.095)	0.04 (0.002 – 1.15) (0.061)
Asthma						
Yes	n/a	n/a	3.55 (0.12 – 105.8) (0.47)	n/a (0.55)	n/a	n/a
No	0.05 (0.003 – 0.83) (0.037)	n/a	0.73 (0.50 – 1.07) (0.11)	0.66 (0.46 – 0.94) (0.021)	0.07 (0.004 – 1.15) (0.063)	n/a
COPD						
Yes	1.22 (0.03 – 48.2) (0.91)	0.28 (n/a) (0.97)	1.22 (0.03 – 48.2) (0.91)	6.69 (0.31 – 145.6) (0.23)	1.00 (0.01 – 92.4) (1.00)	0.01 (n/a) (0.87)
No	0.05 (0.003 – 0.85) (0.038)	0.02 (0.00 – 0.69) (0.031)	0.75 (0.51 – 1.09) (0.13)	0.64 (0.45 – 0.92) (0.016)	0.07 (0.004 – 1.13) (0.061)	0.04 (0.002 – 0.86) (0.04)
Other respiratory diseases						
Yes	1.00 (0.02 – 40.3) (1.00)	0.01 (n/a) (0.67)	0.50 (0.03 – 9.46) (0.64)	0.27 (0.00 – 759.0) (0.75)	2.33 (0.03 – 182.9) (0.70)	n/a (0.99)
No	0.05 (0.003 – 0.85) (0.038)	0.03 (0.001 – 0.54) (0.018)	0.76 (0.52 – 1.11) (0.15)	0.67 (0.47 – 0.96) (0.029)	0.07 (0.004 – 1.13) (0.061)	0.04 (0.002 – 0.88) (0.041)
Cardiovascular diseases						
Yes	0.51 (0.02 – 12.9) (0.68)	0.01 (n/a) (0.82)	0.18 (0.02 – 1.88) (0.15)	0.11 (0.01 – 1.67) (0.11)	2.07 (0.06 –66.3) (0.68)	0.38 (n/a) (0.97)
No	0.05 (0.003 – 0.87) (0.04)	0.03 (0.002 – 0.50) (0.015)	0.76 (0.52 – 1.12) (0.17)	0.70 (0.49 – 0.99) (0.048)	0.07 (0.004 – 1.15) (0.063)	0.04 (0.002 – 0.84) (0.038)
Cancer						
Yes	1.53 (0.05 – 49.8) (0.81)	0.04 (n/a) (0.92)	0.59 (0.02 – 16.7) (0.76)	0.01 (n/a) (0.72)	2.60 (0.04 – 170.4) (0.65)	n/a (0.95)
No	0.05 (0.003 – 0.85) (0.038)	0.01 (0.00 – 0.92) (0.046)	0.76 (0.52 – 1.11) (0.15)	0.68 (0.48 – 0.97) (0.032)	0.07 (0.004 – 1.13) (0.061)	0.04 (0.002 – 0.84) (0.038)
Smoking						
Yes	4.43 (0.15 – 130.1) (0.39)	0.08 (n/a) (0.89)	0.66 (0.03 – 16.8) (0.80)	0.092 (0.00 – 338.6) (0.57)	6.71 (0.11 – 396.2) (0.36)	n/a (0.99)
No	0.05 (0.003 – 0.84) (0.038)	0.03 (0.002 – 0.49) (0.014)	0.76 (0.52 – 1.11) (0.15)	0.68 (0.48 – 0.97) (0.035)	0.07 (0.004 – 1.12) (0.06)	0.04 (0.002 – 0.83) (0.037)
History of stroke						
Yes	0.71 (0.02 – 22.3) (0.85)	0.02 (n/a) (0.82)	0.31 (0.01 – 7.69) (0.47)	n/a (0.73)	2.33 (0.03 – 182.9) (0.70)	n/a (0.97)
No	0.05 (0.003 – 0.87) (0.039)	0.02 (0.00 – 0.58) (0.022)	0.77 (0.53 – 1.13) (0.18)	0.69 (0.48 – 0.98) (0.038)	0.07 (0.004 – 1.13) (0.06)	0.04 (0.002 – 0.84) (0.038)
History of MI						
Yes	n/a	n/a	0.50 (0.02 – 11.1) (0.66)	0.17 (0.01 – 5.14) (0.31)	n/a	n/a
No	0.05 (0.003 – 0.86) (0.039)	n/a	0.74 (0.51 – 1.09) (0.13)	0.69 (0.48 – 0.97) (0.035)	0.07 (0.004 – 1.15) (0.063)	n/a

Appendix 1B: mortality rates in non-users, irregular users, and regular users before matching

Tables 11, 12 and Figure 8 describe mortality rates before matching in ivermectin non-users, irregular users, and regular users. Mortality rate was 2.6% in ivermectin non-users (79/3,034 deaths), 1.9% in irregular users (29/1,542 deaths), and 0.7% in regular users (2/283 deaths). Regular use of ivermectin was associated with 85% reduction in mortality rate when compared to non-use of ivermectin (RR: 0.15; 95% CI: 0.04 - 0.59; p = 0.007) and 77% reduction when compared to irregular users (RR: 0.23; 95% CI: 0.06 - 0.94; p = 0.031). Irregular use of ivermectin was associated with 36% reduction in mortality rate compared to non-use (RR: 0.64; 95% CI: 0.43 - 0.96; p = 0.041).

**Table 11:** Pre-matching mortality rates in overall and in subpopulations among ivermectin non-users, irregular users, and regular users MI = myocardial infarction; COPD = chronic obstructive pulmonary disease; y/o = years old; CI = confidence interval; n/a = not applicable.

Mortality rates	Non-matched (pre-matching) groups				Overall p-values	
Population	Overall (n = 4,859)	Non-users - deaths (%) (n = 3,034)	Regular users - deaths (%) (n = 283)	Irregular users - deaths (%) (n = 1,542)	Unadjusted overall p-value (between three groups)	Unadjusted overall p-value (p) and risk ratio (95% CI) between subpopulations
Overall	110/4,859 (2.3%)	79/3,034 (2.6%)	2/283 (0.7%)	29/1,542 (1.9%)	0.058	
Age						<0.0001
<30 y/o	1/1,280 (0.1%)	1/844 (0.1%)	0/39 (0.0%)	0/397 (0.0%)	n/a	
30-50 y/o	12/2,488 (1.2%)	10/1,582 (0.6%)	0/131 (0.0%)	2/775 (2.6%)	0.05	6.20 (0.81 – 47.7) (0.08) vs < 30 y/o
>50 y/o	97/1,091 (8.9%)	68/608 (11.1%)	2/113 (1.8%)	27/370 (7.3%)	0.002	13.8 (1.92 – 99.0) (0.009) vs < 30 y/o / 2.25 (1.22 – 4.06) (0.009) vs 30-50 y/o
Sex						1.36 (0.93 – 1.99) (0.11) vs female
Female	51/2,618 (1.9%)	36/1,624 (2.2%)	0/141 (0.0%)	15/853 (1.8%)	<0.0001	
Male	59/2,241 (2.6%)	43/1,410 (3.1%)	2/142 (1.4%)	14/689 (2.0%)	0.25	
Race						0.54
Afro-Brazilian	4/141 (2.8%)	4/100 (4.0%)	0/4 (0.0%)	0/37 (0.0%)	0.001	1.18 (0.43 – 3.26) (0.75) vs Caucasians / 1.68 (0.56 – 5.01) (0.35) vs mixed / 1.28 (0.23 – 7.16) (0.78) vs Asian-Brazilian
Mixed	19/1,113 (1.7%)	12/682 (1.8%)	0/58 (0.0%)	7/373 (1.9%)	0.4	0.70 (0.42 – 1.16) (0.17) vs Caucasians / 0.76 (0.18 – 3.33) (0.72) vs Asian-Brazilian
Caucasian	85/3,515 (2.4%)	62/2,192 (2.8%)	2/221 (0.9%)	21/1,102 (1.9%)	0.085	1.09 (0.26 – 4.50) (0.90) vs Asian-Brazilian
Asian-Brazilian	2/90 (2.2%)	1/60 (1.7%)	0/0 (0.0%)	1/30 (3.3%)	0.31	
Type 2 diabetes						11.2 (6.64 – 19.0) (<0.0001)
Yes	20/112 (17.9%)	16/63 (25.4%)	1/9 (11.1%)	3/40 (7.5%)	0.059	
No	90/4,747 (1.9%)	63/2,971 (2.1%)	1/274 (0.4%)	26/1,502 (1.7%)	0.11	
Hypertension						8.79 (5.79 – 13.4) (<0.0001)
Yes	36/285 (12.6%)	28/166 (16.9%)	1/23 (4.3%)	7/96 (7.3%)	0.037	
No	74/4,574 (1.6%)	51/2,868 (1.8%)	1/260 (0.4%)	22/1,446 (1.5%)	0.22	
Asthma						8.76 (1.90 – 40.4) (0.005)
Yes	2/12 (16.7%)	1/6 (16.7%)	0/0 (0.0%)	1/6 (16.7%)	0.91	
No	108/4,837 (2.2%)	78/3,028 (2.6%)	2/283 (0.7%)	28/1,536 (18.2%)	0.054	
COPD						6.21 (0.76 – 51.0) (0.088)
Yes	1/8 (12.5%)	1/6 (16.7%)	0/1 (0.0%)	0/1 (0.0%)	n/a	
No	109/4,851 (2.2%)	78/3,028 (2.6%)	2/282 (0.7%)	29/1,541 (1.9%)	0.065	
Other respiratory diseases						5.44 (0.67 – 43.8) (0.11)
Yes	1/9 (11.1%)	1/5 (20.0%)	0/1 (0.0%)	0/3 (0.0%)	n/a	
No	109/4,850 (2.2%)	77/3,029 (2.6%)	2/282 (0.7%)	29/1,539 (1.9%)	0.074	
Cardiovascular diseases						8.05 (3.05 – 21.2) (<0.0001)
Yes	5/33 (15.2%)	2/15 (13.3%)	0/2 (0.0%)	0/16 (0.0%)	0.001	
No	105/4,836 (2.2%)	77/3,019 (2.6%)	2/281 (0.7%)	29/1,526 (1.9%)	0.078	
Cancer						2.28 (0.30 – 17.2) (0.42)
Yes	1/20 (5.0%)	1/12 (8.3%)	0/2 (%)	0/6 (0.0%)	0.032	
No	109/4,839 (2.2%)	78/3,022 (2.6%)	2/281 (0.7%)	29/1,536 (1.9%)	0.066	
Smoking						0.59 (0.08 – 4.33) (0.61)
Yes	1/73 (1.4%)	1/47 (2.1%)	0/3 (0.0%)	0/23 (0.0%)	0.89	
No	109/4,786 (2.2%)	78/2,987 (2.6%)	2/280 (0.7%)	29/1,519 (1.9%)	0.064	
History of stroke						12.1 (3.32 – 43.9) (0.0002)
Yes	3/14 (21.4%)	4/10 (40.0%)	0/1 (0.0%)	0/3 (0.0%)	0.001	
No	107/4,845 (2.2%)	75/3,024 (2.5%)	2/282 (0.7%)	29/1,539 (1.9%)	0.093	
History of MI						33.3 (7.35 – 150.4) (<0.0001)
Yes	3/7 (42.9%)	1/4 (25.0%)	0/0 (0.0%)	0/3 (0.0%)	n/a	
No	107/4,852 (2.2%)	78/3,030 (2.6%)	2/283 (0.7%)	29/1,539 (1.9%)	0.065	

**Table 12:** Head-to-head comparisons of mortality rates between ivermectin non-users and regular users, non-users and sporadic users, and between sporadic and regular users MI = myocardial infarction; COPD = chronic obstructive pulmonary disease; y/o = years old; CI = confidence interval; n/a = not applicable.

Mortality rates	Non-users versus regular users		Non-users versus irregular users		Regular users versus irregular users	
Population	Unadjusted mortality risk ratio (95% CI) and p-value (p)	Multivariate adjusted mortality risk ratio (95% CI) and p-value (p)	Unadjusted mortality risk ratio (95% CI) and p-value (p)	Multivariate adjusted mortality risk ratio (95% CI) and p-value (p)	Unadjusted mortality risk ratio (95% CI) and p-value (p)	Multivariate adjusted mortality risk ratio (95% CI) and p-value (p)
Overall	0.27 (0.07 – 1.09) (0.066)	0.15 (0.04 – 0.59) (0.007)	0.72 (0.47 – 1.10) (0.13)	0.64 (0.43 – 0.96) (0.041)	0.36 (0.09 – 1.51) (0.16)	0.23 (0.06 – 0.94) (0.031)
Age						
<30 y/o	7.11 (0.29 – 177.5) (0.23)	1.02 (0.00 – 1069.7) (0.99)	0.71 (0.03 – 17.4) (0.83)	0.49 (0.01 – 18.4) (0.70)	10.2 (0.20 – 505.9) (0.24)	1.30 (0.00 – 4060.2) (0.95)
30-50 y/o	0.57 (0.03 – 9.77) (0.70)	0.33 (0.01 – 13.5) (0.56)	0.41 (0.09 – 1.86) (0.25)	0.47 (0.12 – 1.84) (0.28)	1.21 (0.06 – 25.1) (0.90)	0.53 (0.01 – 34.1) (0.76)
>50 y/o	0.14 (0.03 – 0.59) (0.007)	0.15 (0.04 – 0.64) (0.01)	0.63 (0.39 – 0.997) (0.048)	0.68 (0.45 – 1.03) (0.071)	0.24 (0.06 – 0.98) (0.046)	0.24 (0.04 – 1.05) (0.058)
Sex						
Female	0.15 (0.01 – 2.52) (0.19)	0.08 (0.004 – 1.47) (0.089)	0.79 (0.43 – 1.45) (0.45)	0.70 (0.40 – 1.24) (0.22)	0.19 (0.01 – 3.13) (0.24)	0.10 (0.004 – 2.59) (0.17)
Male	0.45 (0.11 – 1.89) (0.28)	0.20 (0.04 – 0.99) (0.048)	0.66 (0.36 – 1.21) (0.18)	0.59 (0.34 – 1.03) (0.065)	0.67 (0.15 – 2.89) (0.59)	0.42 (0.10 – 1.86) (0.26)
Race						
Afro-Brazilian	2.38 (0.11 – 51.4) (0.58)	n/a	0.29 (0.02 – 5.44) (0.41)	0.05 (0.00 – 36.0) (0.36)	4.80 (0.11 – 214.5) (0.42)	n/a
Mixed	0.46 (0.03 – 7.84) (0.59)	n/a	1.07 (0.42 – 2.74) (0.89)	0.76 (0.31 – 1.86) (0.55)	0.42 (0.02 – 7.31) (0.55)	n/a
Caucasian	0.31 (0.08 – 1.29) (0.11)	n/a	0.67 (0.40 – 1.11) (0.11)	0.64 (0.40 – 1.01) (0.058)	0.45 (0.11 – 1.89) (0.28)	n/a
Asian-Brazilian	n/a	n/a	2.03 (0.13 – 33.7) (0.62)	1.81 (0.13 – 24.5) (0.65)	n/a	n/a
Type 2 diabetes						
Yes	0.37 (0.04 – 3.17) (0.36)	0.46 (0.07 – 3.27) (0.44)	0.24 (0.06 – 0.88) (0.031)	0.42 (0.14 – 1.33) (0.14)	1.56 (0.18 – 13.3) (0.69)	0.90 (0.11 – 7.59) (0.92)
No	0.17 (0.02 – 1.22) (0.078)	0.12 (0.02 – 0.63) (0.012)	0.81 (0.51 – 1.29) (0.38)	0.70 (0.45 – 1.08) (0.11)	0.20 (0.03 – 1.48) (0.12)	0.17 (0.03 – 1.03) (0.054)
Hypertension						
Yes	0.22 (0.03 – 1.73) (0.15)	0.24 (0.03 – 1.71) (0.15)	0.39 (0.16 – 0.93) (0.033)	0.48 (0.22 – 1.01) (0.054)	0.54 (0.07 – 4.13) (0.56)	0.53 (0.07 – 4.29) (0.95)
No	0.21 (0.03 – 1.55) (0.13)	0.11 (0.01 – 0.76) (0.025)	0.85 (0.52 – 1.41) (0.54)	0.75 (0.46 – 1.20) (0.23)	0.25 (0.03 – 1.84) (0.17)	0.20 (0.03 – 1.19) (0.077)
Asthma						
Yes	3.67 (0.05 – 274.5) (0.56)	n/a	1.00 (0.05 – 20.8) (1.00)	4.34 (0.04 – 446.1) (0.53)	2.67 (0.23 – 30.4) (0.43)	n/a
No	0.27 (0.07 – 1.10) (0.068)	n/a	0.70 (0.45 – 1.09) (0.11)	0.63 (0.42 – 0.94) (0.024)	0.37 (0.09 – 1.56) (0.18)	n/a
COPD						
Yes	1.22 (0.03 – 48.2) (0.91)	0.92 (n/a) (1.00)	1.22 (0.03 – 48.2) (0.91)	4.98 (0.34 – 73.9) (0.24)	0.50 (0.04 – 7.10) (0.61)	0.03 (n/a) (0.89)
No	0.27 (0.07 – 1.11) (0.069)	0.15 (0.04 – 0.61) (0.007)	0.73 (0.47 – 1.12) (0.14)	0.63 (0.34 – 094) (0.024)	0.38 (0.09 – 1.57) (0.18)	0.27 (0.07 – 1.04) (0.058)
Other respiratory diseases						
Yes	1.00 (0.02 – 40.3) (1.00)	0.04 (n/a) (0.48)	0.43 (0.01 – 14.1) (0.63)	0.13 (n/a) (0.78)	2.0 (0.06 – 68.5) (0.70)	0.26 (0.07 – 1.03) (0.054)
No	0.27 (0.07 – 1.11) (0.069)	0.17 (0.05 – 0.62) (0.01)	0.74 (0.48 – 1.13) (0.16)	0.65 (0.44 – 0.87) (0.037)	0.36 (0.09 – 1.51) (0.16)	0.98 (n/a) (1.00)
Cardiovascular diseases						
Yes	1.08 (0.04 – 30.0) (0.96)	0.03 (n/a) (0.80)	0.16 (0.01 – 3.71) (0.26)	0.02 (n/a) (0.41)	5.67 (0.14 – 234.5) (0.36)	0.61 (n/a) (0.98)
No	0.27 (0.07 – 1.12) (0.072)	0.16 (0.04 – 0.62) (0.008)	0.74 (0.48 – 1.14) (0.17)	0.69 (0.46 – 1.02) (0.061)	0.36 (0.09 – 1.50) (0.16)	0.26 (0.07 – 1.03) (0.056)
Cancer						
Yes	1.53 (0.05 – 49.8) (0.81)	0.3 (n/a) (0.95)	0.59 (0.02 – 16.7) (0.76)	0.01 (n/a) (0.74)	2.33 (0.06 – 92.4) (0.65)	14.7 (n/a) (0.92)
No	0.27 (0.07 – 1.11) (0.069)	0.16 (0.04 – 0.61) (0.008)	0.73 (0.47 – 1.12) (0.15)	0.66 (0.44 – 0.98) (0.039)	0.36 (0.09 – 1.51) (0.16)	0.26 (0.07 – 1.03) (0.055)
Smoking						
Yes	4.43 (0.15 – 130.1) (0.39)	0.33 (n/a) (0.92)	0.66 (0.03 – 16.8) (0.80)	0.12 (0.00 – 272.7) (0.59)	6.25 (0.14 – 272.5) (0.23)	1.69 (n/a) (0.97)
No	0.27 (0.07 – 1.10) (0.067)	0.15 (0.04 – 0.61) (0.008)	0.73 (0.47 – 1.12) (0.15)	0.66 (0.44 – 0.98) (0.04)	0.36 (0.09 – 1.50) (0.16)	0.27 (0.07 – 1.03) (0.056)
History of stroke						
Yes	0.48 (0.02 – 14.7) (0.68)	0.005 (0.00 - > 1000) (0.64)	0.21 (0.01 – 5.05) (0.33)	0.002 (n/a) (0.71)	2.00 (0.06 – 68.5) (0.70)	1.73 (n/a) (0.99)
No	0.28 (0.07 – 1.15) (0.077)	0.20 (0.06 – 0.70) (0.012)	0.76 (0.49 – 1.16) (0.20)	0.68 (0.46 – 1.02) (0.065)	0.36 (0.09 – 1.51) (0.16)	0.26 (0.07 – 1.03) (0.055)
History of MI						
Yes	0.78 (0.02 – 32.4) (0.89)	n/a	0.33 (0.01 – 11.3) (0.54)	0.002 (n/a) (0.74)	4.00 (0.15 – 103.2) (0.40)	n/a
No	0.27 (0.07 – 1.10) (0.068)	n/a	0.73 (0.47 – 1.12) (0.15)	0.67 (0.45 – 0.99) (0.047)	0.36 (0.09 – 1.51) (0.16)	n/a
